# Dynamics of Bacterial Signal Recognition Particle at a Single Molecule Level

**DOI:** 10.3389/fmicb.2021.663747

**Published:** 2021-04-30

**Authors:** Benjamin Mayer, Meike Schwan, Luis M. Oviedo-Bocanegra, Gert Bange, Kai M. Thormann, Peter L. Graumann

**Affiliations:** ^1^LOEWE Center for Synthetic Microbiology, SYNMIKRO, Marburg, Germany; ^2^Department of Chemistry, Philipps Universität Marburg, Marburg, Germany; ^3^Institut für Mikrobiologie und Molekularbiologie, Justus-Liebig-Universität Gießen, Gießen, Germany

**Keywords:** signal recognition particle, protein membrane insertion, *Shewanella putrefaciens*, single molecule tracking, structured illumination imaging

## Abstract

We have studied the localization and dynamics of bacterial Ffh, part of the SRP complex, its receptor FtsY, and of ribosomes in the Gamma-proteobacterium *Shewanella putrefaciens.* Using structured illumination microscopy, we show that ribosomes show a pronounced accumulation at the cell poles, whereas SRP and FtsY are distributed at distinct sites along the cell membrane, but they are not accumulated at the poles. Single molecule dynamics can be explained by assuming that all three proteins/complexes move as three distinguishable mobility fractions: a low mobility/static fraction may be engaged in translation, medium-fast diffusing fractions may be transition states, and high mobility populations likely represent freely diffusing molecules/complexes. Diffusion constants suggest that SRP and FtsY move together with slow-mobile ribosomes. Inhibition of transcription leads to loss of static molecules and reduction of medium-mobile fractions, in favor of freely diffusing subunits, while inhibition of translation appears to stall the medium mobile fractions. Depletion of FtsY leads to aggregation of Ffh, but not to loss of the medium mobile fraction, indicating that Ffh/SRP can bind to ribosomes independently from FtsY. Heat maps visualizing the three distinct diffusive populations show that while static molecules are mostly clustered at the cell membrane, diffusive molecules are localized throughout the cytosol. The medium fast populations show an intermediate pattern of preferential localization, suggesting that SRP/FtsY/ribosome transition states may form within the cytosol to finally find a translocon.

## Importance

Insertion of membrane proteins is mediated by a soluble protein/RNA complex termed “Signal recognition particle,” which recognizes sequences at the N-terminus of proteins during translation, in all types of organisms. The SRP/ribosome nascent chain (NC) complex moves to find the SRP receptor, called FtsY in bacteria, which orchestrates the hand-over of the ribosome NC complex to the integral membrane transporter called translocon, which mediates membrane insertion during continued translation. We have visualized SRP, ribosomes, and FtsY by structured illumination microscopy, and analyzed protein dynamics by single molecule tracking. Our data suggest the existence of three SRP molecule populations, likely representing freely diffusing SRPs, SRP in transition complexes with the ribosome, and static, membrane-associated ribosome NC/SRP/FtsY complexes. Transition and static populations show preferential location in the cytosol and/or exclusively at the membrane, revealing a more intricate spatio-temporal interplay of the three components than was appreciated before.

## Introduction

**FIGURE 1:**
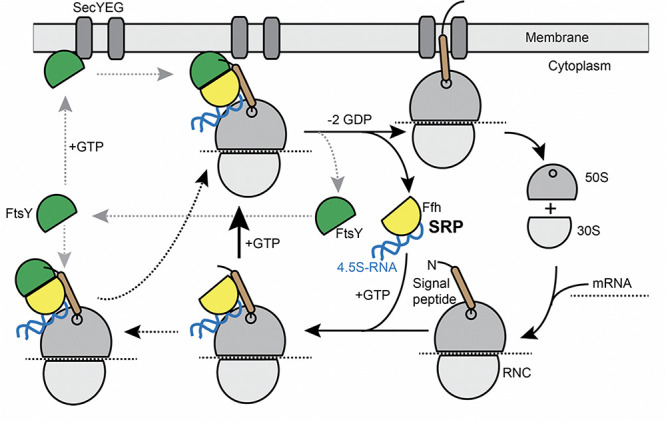
Model for the SRP cycle. Lower part from right to left: ribosome subunits binding to mRNA form the 70S particle, to which SRP binds upon detection of the signal sequence at the nascent chain (“signal peptide”). This complex binds to FtsY, either within the cytosol, as indicated by the leftwards arrow, or at the cell membrane. GTP hydrolysis triggers hand over of the ribosome nascent chain complex to the SecYEG translocon (upper part), upon which SRP and FtsY are released. Our data suggest that complex formation of SRP/RNC and FtsY can occur within the cytosol and also at the membrane.

Transmembrane (TM) proteins comprise at least a quarter of all cellular proteins, and their insertion into the membrane is an essential process for all cells. TM proteins are inserted into the plasma membrane already during their synthesis (co-translational) by the signal recognition particle (SRP) (reviewed in Akopian et al., 2013; Steinberg et al., 2018). SRP is a universally conserved ribonucleo-protein particle, present in all domains of life, whose core consists of the SRP-RNA (termed 4.5S RNA in *Escherichia coli* and scRNA in *Bacillus subtilis*) and the guanosine tri-phosphatases (GTPase) Ffh (Grudnik et al., 2009). SRP recognizes hydrophobic signal sequences present at the N-terminus of nascent TM proteins as soon as they emerge from a translating ribosome (Figure 1). Subsequently, these SRP-bound ribosome nascent chain complexes (RNCs) interact with the SRP receptor GTPase FtsY, which can be membrane-associated via a membrane anchor, in order to guide the RNC onto a vacant SecYEG translocon (Figure 1). After successful transfer of the RNC to the translocon, SRP and its receptor dissociate in order to begin a new round of membrane protein targeting to membranes (Figure 1). Two structurally and functionally conserved GTPase domains present in SRP and FtsY regulate the SRP-cycle through a unique complex in which both GTPases reciprocally stimulate their activities in a shared active site (Egea et al., 2004; Focia et al., 2004; Wild et al., 2016). Both GTPases coordinate the transfer of a hydrophobic signal sequence-presenting RNC onto a vacant translocation channel through a series of precisely orchestrated SRP-RNA-dependent structural rearrangements, which are well understood at atomic resolution (summarized in Saraogi and Shan, 2014). Despite the enormous structural and biochemical understanding of the SRP-pathway over the past decades, in-depth *in vivo* analysis using modern high-resolution fluorescence microscopy and single molecule analysis focusing on the individual steps including the RNC have been lacking to date. In most textbooks, the SRP/ribosome NC complex is depicted to assemble in the cytosol and diffuses to the membrane, where FtsY is encountered to assemble a further complex that then moves to the translocon in a 2D movement along the membrane. On the other hand, several reports have suggestive evidence that mRNA molecules for membrane proteins are localized at the cell membrane (Prilusky and Bibi, 2009; Nevo-Dinur et al., 2011; dos Santos et al., 2012; Libby et al., 2012; Benhalevy et al., 2017). Thus, potentially, SRP could bind to ribosomes that are already membrane-proximal, to quickly find FtsY receptors. The latter protein has been shown to be predominantly membrane-bound, supporting a scenario of complex formation close to or at the cell membrane.

Single molecule tracking not only allows detection and tracking of single molecules at high temporal resolution, such that even freely diffusive proteins can be detected and analyzed, but it also allows determining the position of molecules with high precision. However, data on single molecule dynamics are scarce, although they can be highly powerful to describe spatiotemporal aspects of processes within live cells. In this study, we have visualized the movement of SRP, FtsY, and of ribosomes in *Shewanella putrefaciens* CN32, henceforth called *S. putrefaciens*, with a localization precision of below 50 nm (Dersch et al., 2020). We were able to generate functional fluorescent protein fusions to Ffh, FtsY, and the ribosomal large subunit protein L1, which are inserted at the original gene locus and are driven by the original promoter, generating fusions expressed at physiological levels. Data obtained can be best explained by assuming three populations of molecules with distinct diffusion coefficients for all three proteins/protein complexes. Non-constrained particle diffusion depends on size and can be interpreted based on principles of Brownian motion. The slowest (static) fraction likely represents translating ribosomes as well as ribosome nascent chain complexes together with SRP and FtsY at translocons, while the fastest mobile fractions include freely diffusing subunits. The medium fast fractions are likely transition complexes including SRP/RNA/FtsY complexes in search of translocons, which we localize close to the cell membrane as well as within the cytosol.

## Results

### SRP and FtsY Occupy Predominantly Membrane-Proximal Spaces, but Are Not Accumulated at Polar Sites Like Ribosomes

We wished to investigate the dynamics of SRP-driven integration of membrane protein in live bacteria. We therefore generated a C-terminal fusion of Ffh to monomeric fluorescent protein sfGFP, integrated at the original gene locus in a markerless manner, such that the fusion is the sole source of the protein expressed in the cell and that expression is driven by the native promoter, yielding physiological levels (Supplementary Figure 1). Cells expressing Ffh-sfGFP grew with indistinguishable doubling time as wild type cells, showing that the fusion protein can functionally replace wild type Ffh. We used an analogous strategy to generate functional fusions for FtsY and for L1 protein (encoded by *rplA*), part of the large ribosomal subunit, and both fusions proved to be stable and fully functional as judged from identical doubling times like wild type cells devoid of any fusion construct.

**FIGURE 2:**
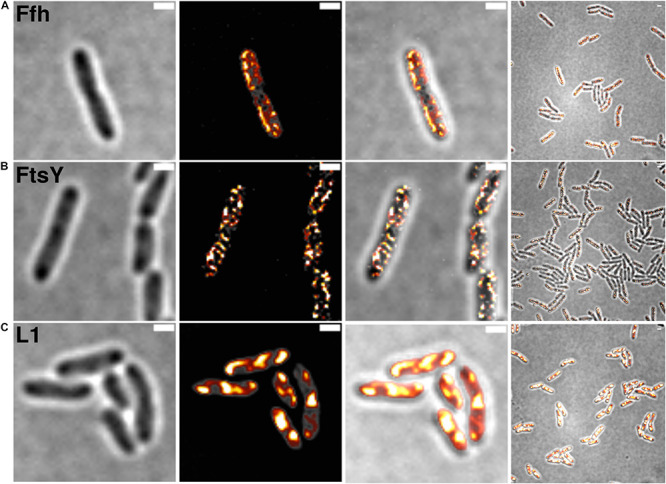
Structured illumination microscopy imaging of SRP interaction partners in *S. putrefaciens* at mid-exponential phase. **(A)** Static fractions of Ffh are distinctly located in close proximity to the cell membrane. Z-stack projection shows localization densities of static Ffh particles. Higher particle densities and gradients are indicated by multi-color coding with gray (little signal) to red-shifted (high signal). **(B)** Static fractions of FtsY also show highest densities close to the cell membrane. **(C)** Different from Ffh and FtsY, L1 shows distinct subcellular localizations representing nucleoid occlusion with high localization density at pole regions and septum. Z-stack projection shows similar patterns throughout the cells. Proteins are fused to sfGFP and are expressed as sole source of the proteins, at physiological levels. Left panels bright field acquisition, second panels SIM acquisition, third panels color coded SIM gradient, right panels color coded Z-stack projections. Scale bars 1 μm.

Figure 2 shows SIM images of cells, where ribosomes are found as large accumulation at the cell poles, in between nucleoids (in the cell center) in large cells, and at other membrane-proximal sites (Figure 2C). This “nucleoid-occlusion” type of localization is known from *E. coli* and from *B. subtilis* cells and shows that *S. putrefaciens* cells have a similar 3D arrangement with regard to their nucleoids and ribosomes. Distinct from ribosomes, Ffh and FtsY revealed predominantly membrane-proximal localization, but no accumulation at the cell poles or in between segregated nucleoids (i.e., the cell center, Figures 2A,B). Thus, visualization of Ffh indicates that the protein is distributed around the entire cell membrane, and similarly its receptor, in a non-homogeneous manner. It follows that integration of membrane proteins occurs all over the cell membrane, in agreement with earlier investigations studying the localization of the SecYEG translocon and of FtsY (Mircheva et al., 2009; Dajkovic et al., 2016; Dempwolff et al., 2016), and is not restricted to the cell poles, as had been suggested for Tat transport (Berthelmann and Bruser, 2004; Rose et al., 2013). Of note, while most ribosomes are engaged in the translation of soluble proteins, approximately 10–15% are membrane-bound, according to cell fractionation (Randall and Hardy, 1977; Herskovits and Bibi, 2000), and thus active in translation of membrane proteins. Thus, SRP is only bound to a subpopulation of ribosomes. It must be kept in mind that SIM is based on slower wide field acquisition; it mostly captures statically positioned molecules, but not mobile and thus freely diffusing molecules. Therefore, clusters of SRP and FtsY at the cell membrane likely consist of static molecules, and nothing can be deduced for freely diffusing molecules, which is likely for any protein or complex. In order to gain a better temporal resolution for analyzing protein dynamics in more detail, we turned to single molecule tracking.

### Ffh, FtsY, and Ribosomes Show Three Distinct Diffusive Populations

**FIGURE 3:**
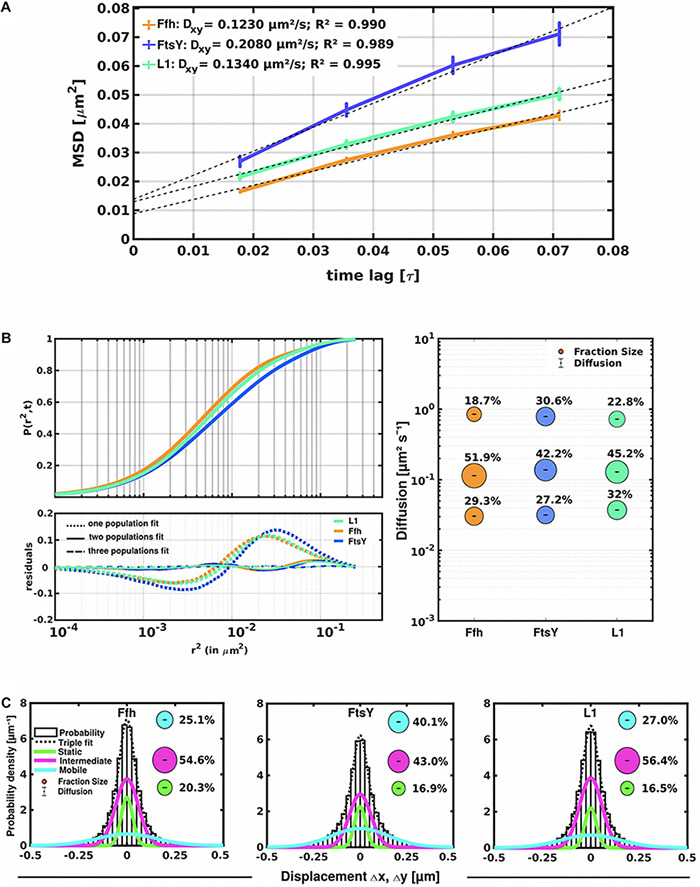
Tracking of SRP, FtsY, and ribosomes in *S. putrefaciens*. **(A)** Mean squared displacement (MSD) analyses of Ffh-mVenus, FtsY-mVenus, and L1-mVenus (abbreviated “mV”), showing different overall diffusion constants, corresponding to each molecular weight. MSD assumes an overall diffusion regardless of subdiffusion. **(B)** Squared displacement (SQD) analyses shows measured data, suggesting three significantly distinct diffusive subpopulations (upper panel). Residual analysis of SQD confirms three population assumption (lower panel): colored curves represent differences between measured data and modeled data (using Brownian motion), which are represented by the “zero” line. Right panel shows a bubble blot, illustrating mean diffusion constants (y-axis) of fractions indicated as circles, with sizes corresponding to fraction size (also written above the circle). **(C)** Gaussian mixture model (GMM) analyses also identify three significantly distinct diffusive subpopulations at steady-state. Bubbles illustrate fraction sizes, with fractions indicated by the different colored lines. Dashed line indicates sum of the three GMM fits.

Freely diffusive proteins such as GFP or monomeric fluorescent proteins can only be accurately followed by very fast acquisition rates (e.g., 5 ms; Stracy et al., 2014; Schibany et al., 2018), because rapid movement during longer acquisition times leads to blurring of the signal. Because we wanted to analyze movement of larger proteins (FtsY) and of protein/RNA complexes, we used an acquisition rate of 16 ms, which is able to capture movement of freely diffusive larger proteins (Sanamrad et al., 2014), but can also quantify diffusion constants of slow-mobile particles. We used YFP-bleaching single molecule/particle tracking (SMT) on strains carrying fluorescent protein fusions, which avoids blue light toxicity, and employed laser power density of about 160 W cm^–2^ that allows for continued growth of cells after acquisition (Supplementary Movie 1). In brief, the central part of a 514 nm laser is focused on the back focal plane of the objective, and when most molecules are bleached in the cells (this can be determined by analyzing bleaching curves obtained in “ImageJ/Fiji”), single molecules can be tracked in real time (Reyes-Lamothe et al., 2010; Hernandez-Tamayo et al., 2019). Many hundreds of cells (Table 1) are imaged for each experiment to obtain a sufficient number of tracks. When we analyzed mean squared displacement (MSD) of the protein fusions, which determines movement averaged over many steps taken by the molecule, we found Ffh to move slowest, with 0.123 μm^2^/s, and FtsY to move fastest, with 0.208 μm^2^/s, while ribosomes showed an intermediate diffusion constant of 0.134 μm^2^/s (Figure 3A). This is somewhat surprising as ribosomes are by far the largest particles among the three proteins/complexes analyzed. However, MSD analyses determine one average diffusion coefficient for all molecules detected (only molecules showing continuous signals for at least 5 steps were taken into account). In order to determine if several populations with different diffusion properties exist, we employed squared displacement (SQD) analyses, in which the probability of molecules moving with a certain displacement in x and y direction (squared) is scored. All single molecule data analyses were done using SMTracker (Rosch et al., 2018), which uses R^2^ as a measure for goodness and several statistical tests (Kolmogorov-Smirnov-Goodness-of-Fit, null hypothesis significance) to determine if acquired distributions of molecule movements can be explained by single or multiple fractions. Figure 3B recapitulates MSD analysis in that overall movement of Ffh molecules was lowest, and that of FtsY highest, with larger displacement being toward the right on the x-axis. The lower panel of Figure 3B shows the differences (colored curves, “residuals”) between measured values and data modeled according to random diffusion, represented by the “0” line.” Assuming that only one population of diffusive Ffh molecules exists (i.e., using a single fit to explain the data) leads to a strong deviation between the modeled data and the determined displacements (dotted curves), while fitting the data assuming two populations having distinct diffusion constants brings the expected values closer to the *in vivo* data (solid lines), but deviations still clearly exist. Using three fits for three populations yielded fewer residuals than two populations (dashed lines) to explain the experimental data, indicating that likely, three distinct populations exist for all three protein fusions. Importantly, the R^2^ factor for three populations was 1 (Table 1). This finding indicates that the assumption of four or more fractions would result in overfitting of the data, and was therefore rejected as a plausible possibility. SMTracker uses Bayesian Information Criterion (BIC) to avoid over-fitting of data, which in case of single molecule movement of Ffh, FtsY, and L1 is important to make sure that the movement of the three proteins assuming three distinct populations rather than two fractions is not a mathematical artifact. Figure 3B (right panel) shows the determined diffusion constants on the y-axis, and the size of the populations as reflected by the size of the circles. According to SQD analysis, 29.3 ± 0.001% of Ffh-mVenus molecules were in a slow diffusive/static mode, with a diffusion coefficient of 0.03 ± 0.001 μm^2^/s (Table 1), 52 ± 0.001% of molecules showed an intermediate average diffusion coefficient of 0.11 ± 0.001 μm^2^/s, and 19 ± 0.001% comprised the fast-mobile fraction with D = 0.85 ± 0.002 μm^2^/s (Table 1), likely representing free SRP complex. In order to learn more about the nature of the different populations observed for SRP/Ffh, we determined dynamics of ribosomes, according to SQD analyses. We found that 32% of L1 molecules were in a slow diffusive/static mode (0.037 ± 0.001 μm^2^/s, Table 1). This fraction most likely represented actively translating 70S ribosomes, which in *E. coli* have been determined to have a comparable diffusion constant of 0.055 μm^2^/s, by the Elf group (Sanamrad et al., 2014). A second population of 45% molecules we detected moved with a medium diffusion rate of 0.129 ± 0.001 μm^2^/s (Table 1), while the third detected one of 23% had a diffusion constant of 0.72 ± 0.001 μm^2^/s (Figure 3B and Table 1). Free large or small ribosomal subunits were measured by SMT to move with 0.4 μm^2^/s (Sanamrad et al., 2014), which more closely resembles the fast fraction. Additionally, the ratio of free subunits vs. actively translating ribosomes has been determined to be 15–85% by Forchhammer and Lindahl (1971). Therefore, we favor the view that the fast fraction of about 23% of L1 molecules corresponds to free large 50S subunits. The medium-mobile fraction for L1 we observed must be composed of particles considerably larger than free subunits, but smaller than translating ribosomes and polysomes. We suggest that this population is composed of transition complexes, including 70S/mRNA initiation complexes, on the move toward the cell periphery or the cell poles. Here, actively translating polysomes on mRNA, as observed by epifluorescence (Lewis et al., 2000; Mascarenhas et al., 2001) and SIM (Figure 2), likely comprise the static fraction.

**TABLE 1:** Squared displacement analyses.

	**Ffh-mVenus**	**FtsY-mVenus**	**L1-mVenus**
# Movies	26	21	19
# Cells	144	218	109
Av. cell length (μm)	2.4400	2.4000	2.7800
# Tracks	7,010	5,269	22,706
Av. lifetime (frames/s)	7/0.12	6.8/0.12	8.5/0.15
pop_1_ (%)*	29.3 ± 0.001	27.2 ± 0.001	32 ± 0
pop_2_ (%)	51.9 ± 0.001	42.2 ± 0.001	45.2 ± 0
pop_3_ (%)	18.7 ± 0	30.6 ± 0.001	22.8 ± 0
D_1_ (μm^2^ s^−1^)	0.0304 ± 0	0.0317 ± 0	0.0372 ± 0
D_2_ (μm^2^ s^−1^)	0.114 ± 0	0.137 ± 0	0.129 ± 0
D_3_ (μm^2^ s^−1^)	0.85 ± 0.002	0.79 ± 0.001	0.72 ± 0.001
Pearson R-squared			
R^2^ (1 frame)	1	1	1
Best model	Triple fit	Triple fit	Triple fit

*pop = size of population with corresponding diffusion constant D.

For FtsY-mVenus, we determined diffusion constants of 0.032 μm^2^/s for 27% of molecules, 42% of molecules being in a medium-mobile mode with D = 0.14 μm^2^/s, and 0.79 μm^2^/s for the fast population of 31% (Figure 3B and Table 1). Thus, slow mobile/static Ffh/SRP has a similarly low diffusion constant as the slow mobile/static fraction of FtsY and of putatively actively translating ribosomes/polysomes. Given the fact that we cannot distinguish between polysomes translating soluble proteins and ribosomes translating membrane proteins at the Sec translocon, we propose that the slow mobile/static fractions of SRP and of FtsY are engaged with the latter fraction of ribosomes.

The intermediate-mobile populations of Ffh/SRP and of FtsY have a similar diffusion coefficient to that observed for intermediate-mobile L1 (Figure 3B and Table 1). These findings are compatible with SRP and FtsY moving together with some ribosomes from the intermediate-mobile fraction of ribosomes, representing SRP/FtsY/ribosome NC complexes. Please note that we have been using movement captured in x/y direction to calculate diffusion constants. As movement in the cell occurs in 3D, our determined numbers are somewhat underestimates of true diffusion constants *in vivo*. Also, movement of membrane, or membrane-attached proteins, is underestimated by a factor of 1.3 (Lucena et al., 2018) because of membrane curvature, so a fraction of molecules (e.g., for FtsY having an amphipathic membrane-tethering helix and the SecYEG interacting A-domain; Angelini et al., 2005) will have a higher diffusion constant *in vivo* than we have determined; however, this does not considerably compromise our estimates for protein dynamics.

To further test for the presence of three distinct populations using another type of data analysis, we used Gaussian mixture modeling (GMM), where the probability of molecules taking a certain length of steps (rather than the squared displacement as in SQD) is scored. We have shown that GMM and SQD perform almost identically well with modeled data (Rosch et al., 2018). Using GMM, we also obtained best fits using three populations to explain the experimental data (Figure 3C and Table 2) (R^2^ values of 1). Kolmogorow-Smirnow goodness-of-fit (KS GoF) testing (implemented in SMTracker 1.5; Kunz et al., 2020) rejected fitting using a single or two fits, while that using three fits was accepted (Table 2). While 20% of Ffh, 17% of FtsY, and 17% of ribosomes were in a slow mobile/static mode, 56% of ribosomes, 55% of Ffh, and 43% of FtsY moved at the intermediate motion, while 27, or 25, or 40% were freely diffusive (Figure 3C). As explained above, diffusion constants are different from those determined by SQD (Table 1), because the same distinct (3) constants are determined from fitting of data for the three proteins that can best explain all steps observed, and then population sizes are deduced. Because it is easier to compare changes in dynamics by analyzing differences in population sizes between proteins and between different conditions, we used GMM for further analysis.

**TABLE 2:** Gaussian mixture modeling analyses.

**Stars/*p*-value**	**Ffh-mVenus**	**FtsY-mVenus**	**L1-mVenus**
**Single fit**			
D ± SD (μm^2^ s^−1^)	0.12 ± 0.00036	0.13 ± 0.00044	0.13 ± 0.00019
K-S GoF* test	Rejected	Rejected	Rejected
*P*-value	0	0	0
R-squared	0.997	0.992	0.997
**Double fit**			
Static D ± SD (μm^2^ s^−1^)	0.054 ± 6.4e−05	0.054 ± 6.4e−05	0.054 ± 6.4e−05
Mobile D ± SD (μm^2^ s^−1^)	0.47 ± 0.00093	0.47 ± 0.00093	0.47 ± 0.00093
Static fraction ± SD (%)	63 ± 0.061	48 ± 0.067	59 ± 0.06
Mobile fraction ± SD (%)	37 ± 0.061	52 ± 0.067	41 ± 0.06
K-S GoF test	Rejected	Rejected	Rejected
*P*-value	0.005	0.0009	4.1e−05
R-squared	1	1	1
**Triple fit**			
Static D ± SD (μm^2^ s^−1^)	0.025 ± 0.00011	0.025 ± 0.00011	0.025 ± 0.00011
Slow-mobile D ± SD (μm^2^ s^−1^)	0.096 ± 0.00028	0.096 ± 0.00028	0.096 ± 0.00028
Mobile D ± SD (μm^2^ s^−1^)	0.66 ± 0.0013	0.66 ± 0.0013	0.66 ± 0.0013
Static fraction ± SD (%)	20 ± 0.14	17 ± 0.11	17 ± 0.13
Slow-mobile fraction ± SD (%)	55 ± 0.11	43 ± 0.095	56 ± 0.093
Mobile fraction ± SD (%)	25 ± 0.13	40 ± 0.1	27 ± 0.11
K-S GoF test	Accepted	Accepted	Accepted
*P*-value	0.43	0.78	0.57
R-squared	1	1	1
Best model	Triple fit	Triple fit	Triple fit

*Kolmogorov-Smirnov Goodness-of-fit Test.

### Ffh and FtsY Mobilities Change in Response to Inhibition of Transcription or of Translation

**FIGURE 4:**
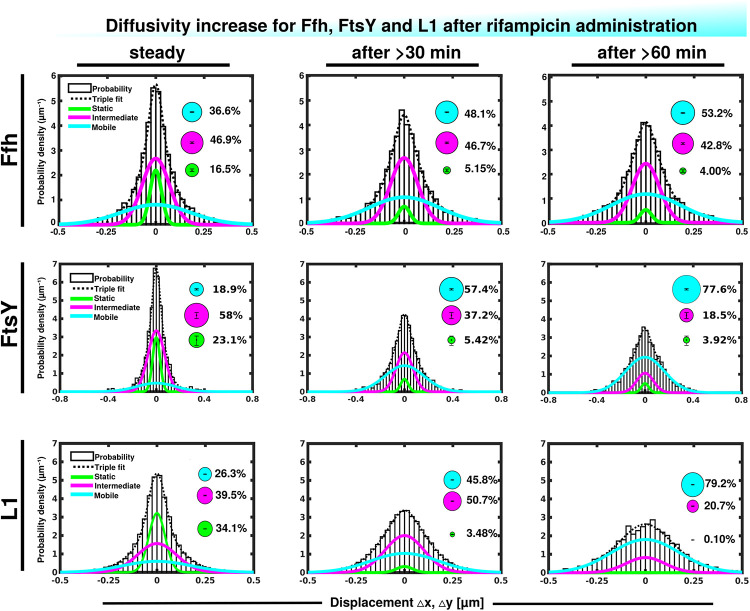
Gaussian mixture model (GMM) analyses of protein dynamics in response to rifampicin treatment, inhibiting transcription (tracking 30 or 60 min after addition of a sublethal concentration of Rif). Population sizes of mVenus fusions and time of imaging after addition of rifampicin are stated in the panels. Bubbles illustrate fraction sizes, fractions are indicated by the different colored lines, analogous to Figure 3C.

We wished to obtain more insight into the nature of the different fractions of the SRP system and therefore treated cells with rifampicin to inhibit mRNA synthesis. Please note that a common diffusion constant (D) was determined via GMM analyses for all three conditions/time points, such that only population sizes differ between the conditions, rather than both diffusion constants and population. This procedure facilitates the comparison between molecules in non-stressed and in stressed cells. Using a joint fitting leads to a change in population sizes relative to data shown in Figure 3C, where “D” was determined only to non-stressed conditions. For Ffh, inhibition of transcription progressively abolished the static fraction, in favor of the fast diffusing fraction, while the medium mobile fraction decreased by about 10% (Figure 4 and Supplementary Table 1). This can be well seen by the reduction of small steps (close to zero) to the favor of larger steps, such that the distribution becomes much wider after drug addition. We interpret this behavior to represent the loss of mRNA substrate binding to ribosomes, also leading to a depletion of SRP binding to ribosomes and abolishment of delivery of ribosome/NCs to the translocon via SRP/FtsY binding, while the medium fast fraction, which may contain SRP/ribosome nascent chain complexes in search for a translocon, is reduced. The increase in the fast mobility fraction of Ffh-mVenus likely represents freely diffusing SRP particles not finding new ribosome/NC complexes.

For FtsY, the static fraction decreased to almost zero, too, and the medium-mobility population also decreased significantly, but a considerable percentage of molecules remained in this state (Figure 4 and Supplementary Table 1). For ribosomes, the static, actively translating fraction disappeared and the medium fast fraction strongly declined (Figure 4), similar to FtsY, and somewhat different to Ffh with regards to the middle fraction. However, it has to be kept in mind that most ribosomes will not be interacting with SRP, and thus changes in SRP dynamics may not necessarily be accompanied by similar effects on all ribosomes. Thus, loss of substrate leads to disassembly of active ribosomes, and also to some degree for the putative SRP/FtsY/70S ribosome complex, which would have been expected.

**FIGURE 5:**
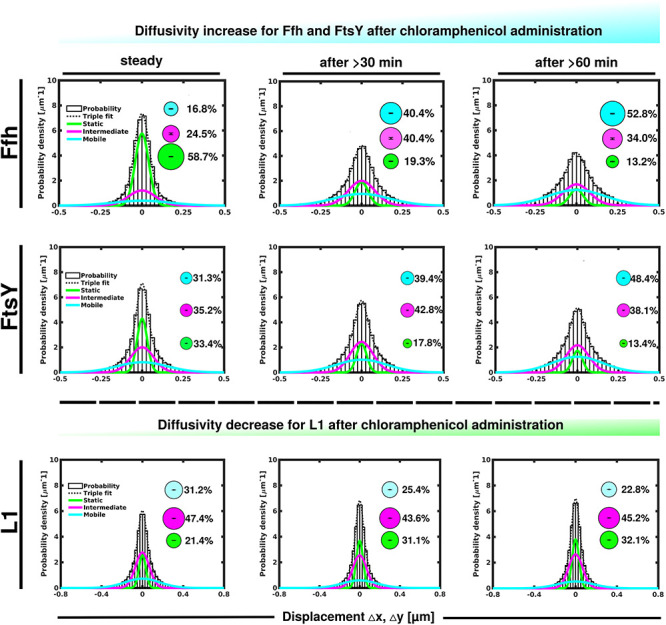
GMM analyses after addition of chloramphenicol, inhibiting translation by blocking peptidyl transferase activity. GMM analysis identifies three significantly distinct diffusive subpopulations indicated by the different colored lines, dashed line shows sum of the three fits. Bubbles illustrate fraction sizes.

Contrary to the inhibition of transcription, stalling of translation via addition of chloramphenicol led to an increase in statically positioned ribosomes (Figure 5), which are unable to finish translation (chloramphenicol blocks the peptidyl transferase center). The medium mobile population remained relatively stable (Supplementary Table 1), while the mobile fraction declined (Figure 5, lower panels). These data suggest that while some 70S ribosomes can still form, active translation complexes are blocked and remain in this static state of mobility.

Markedly different from ribosomes, a reduction in translation elongation led to a decrease of the static fractions of both Ffh and FtsY, and interestingly, first to an increase in the medium fraction after 30 min followed by a decline after 60 min, still resulting in a higher population size compared to steady state conditions (Figure 5 and Supplementary Table 1). Increase in the medium mobility population was most pronounced for Ffh. Thus, stalling of ribosomes has a different effect on ribosomes or on Ffh and FtsY mobility, possibly because 85–90% of ribosomes do not interact with SRP/FtsY.

**FIGURE 6:**
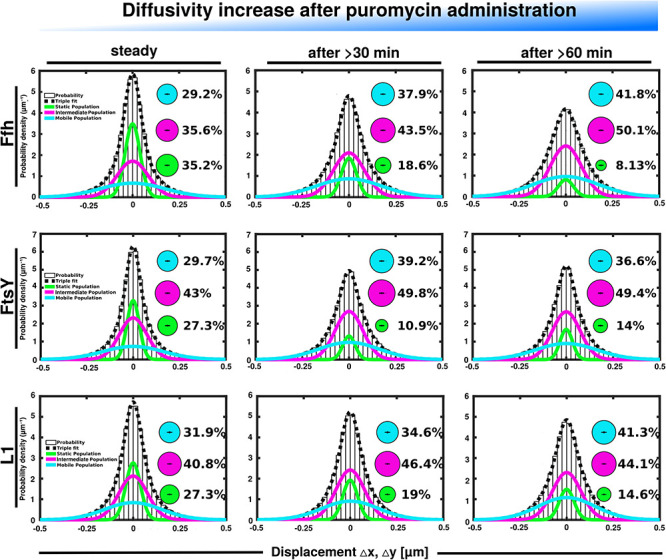
GMM analyses after addition of puromycin, leading to premature translation termination and mRNA release. GMM analysis identifies three significantly distinct diffusive subpopulations indicated by the different colored lines, dashed line shows sum of the three fits. Bubbles illustrate fraction sizes.

Further, we treated cells with puromycin in order to enforce premature termination of translation, accompanied by the release of nascent chains from ribosomes. Based on premature termination events, and thus shorter extension times, we would have expected a loss of statically positioned ribosomes, which indeed was the case, as shown in Figure 6 (lower panels). The number of synthetically active (slow mobile) ribosomes was almost halved 60 min after the addition of puromycin, while the population of freely diffusing subunits strongly increased (Figure 6 and Supplementary Table 1). Note that addition of puromycin led to considerable cell elongation before growth ceased, respective imaging data was further investigated using a customized workflow (Mayer et al., 2021), underscoring the effectiveness of treatment. Interestingly, Ffh became much more dynamic, in that the static fraction was reduced to about 23% of steady state levels (Figure 6, upper panels), and the fast-dynamic fraction strongly increased, indicative of an increase in free SRP particles (Supplementary Table 1). Although puromycin will lead to a reduction in the number of nascent chains (Azzam and Algranati, 1973), the medium mobile population thought to contain a ribosome-bound SRP fraction also increased. We cannot fully explain this observation but speculate that ribosomes may continue to initiate translation for some time and thus engage in SRP binding in case mRNA for membrane proteins has been bound.

For FtsY, changes occurring after addition of puromycin were similar to those seen for SRP, however, they were strongest after 30 min of drug addition, however, they were less pronounced (Supplementary Table 1). However, as for SRP, premature termination led to a strong decrease in the static fraction and an increase in likely freely diffusive molecules (Figure 6, middle panels).

In order to analyze if changes seen for translation inhibition were specific for Ffh, FtsY, and ribosomes, we visualized the dynamics of a protein that is unrelated to translation. We chose the enhancer binding protein FlrA, which plays an important role in gene regulation for establishment of the single polar flagellum in *Shewanella* (Blagotinsek et al., 2020). FlrA is a hexameric AAA^+^ protein that influences RNA polymerase activity (Gao et al., 2020). Supplementary Figure 2 shows that the single molecule steps taken by a functional fusion of FlrA to mVenus can be well explained by assuming two distinct populations, a static population (likely DNA-bound FlrA), and a freely diffusive fraction, i.e., unbound FlrA-mVenus molecules. An R^2^ value of 1 (Supplementary Table 1) indicates that assuming three distinct populations would result in an overfitting of the data. Sixty minutes after addition of puromycin, the percentage of static molecules is increased at the expense of freely diffusing molecules (Supplementary Figure 2). This experiment shows that increased mobility is not a general property of cytosolic proteins in response to translation stress, supporting the idea that Ffh, FtsY, and L1 show translation-specific changes in their behavior, which are different dependent on whether translation is blocked or prematurely terminated.

### Depletion of FtsY Has Only a Small Effect on the Dynamics of Ffh, but Leads to Delocalization of Ffh

We wished to analyze the effect of depletion of FtsY on the mobility of Ffh, wondering how a depletion of FtsY would affect the three mobility populations of SRP. For depletion we employed the L-arabinose utilization gene locus of *S. putrefaciens* CN-32 (Watanabe et al., 2006; Rodionov et al., 2010). To this end, we placed a copy of *ftsY* on the chromosome under tight control of an AraR-controlled promoter, which allowed wild type-level expression in the presence of 0.1% arabinose (Supplementary Figure 3). In this strain, native *ftsY* was removed by an in-frame deletion in the presence of arabinose. Omitting the inducer resulted in FtsY depletion to less than 10% of wild-type levels, resulting in a drastic slow-down of growth (Supplementary Figure 4).

**FIGURE 7:**
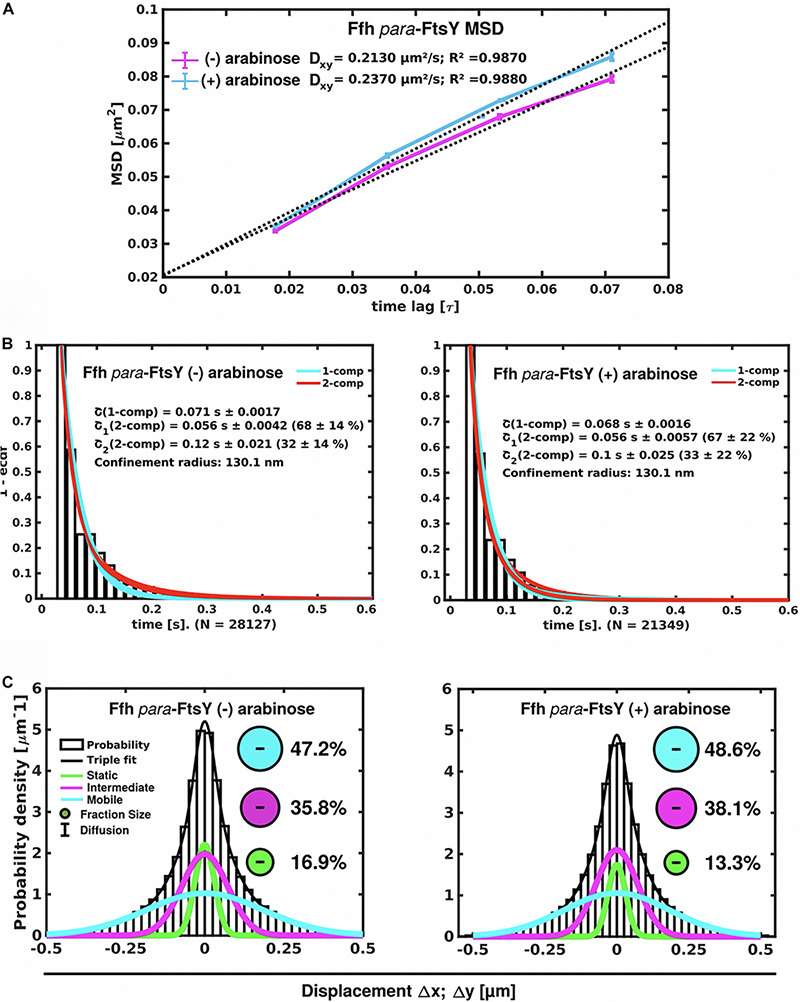
Changes in Ffh dynamics in response to FtsY depletion. **(A)** MSD analysis reveals slightly reduced mobility after depletion of FtsY (“- arabinose”). **(B)** Minor changes in dwell times, determined using a 2 population decay curve, after FtsY depletion (Ffh becomes slightly more static), **(C)** GMM analyses show a small increase in static Ffh molecules after FtsY depletion.

**FIGURE 8:**
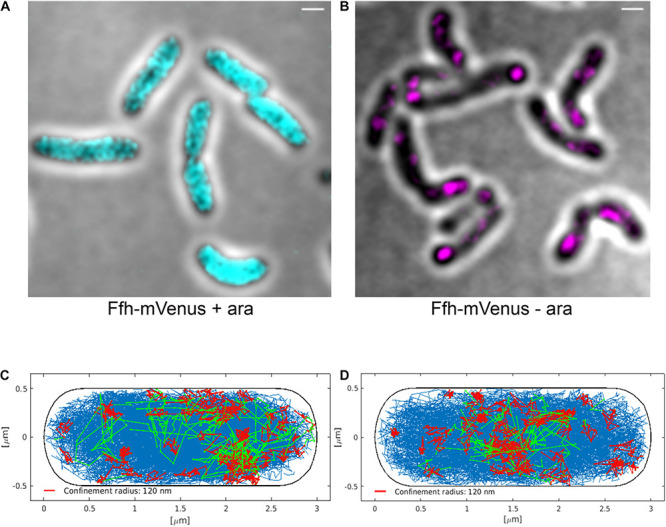
Averaged 3D-SIM image stack projection and confinement maps of Ffh-sfGFP in cells expressing FtsY under control of the arabinose promoter, false-colored in cyan **(A)**, or in cells after depletion of FtsY due to growth in the absence of arabinose, false-colored in magenta **(B)**. Scale bar 1 μm. **(C)** Confinement map of Ffh-mVenus tracks in the presence of arabinose (FtsY), or **(D)** in the absence of arabinose/FtsY. Blue track non-confined motion, red tracks confined motion of at least 9 steps within a radius of 120 nm, green tracks transitions between confined and free motion, projected into a standardized cell of 3 × 1 μm size.

Interestingly, Ffh dynamics did not change considerably. Overall, diffusion of Ffh-mVenus molecules increased in the absence of FtsY (Figure 7A), and dwell times somewhat decreased (Figure 7B). We observed a small decrease in the static population, an increase in the medium velocity population, and an almost unchanged free mobile population (no arabinose vs. FtsY synthesis during presence of arabinose, Figure 7C and Supplementary Table 2). We interpret these data to indicate that SRP can associate with ribosomes and remain bound in the absence (or strongly reduced amount) of FtsY, while release is slowed down. This agrees with the observation that after depletion of FtsY, Ffh localization changed dramatically, in that few remaining structures were visible (Figure 8B and Supplementary Movie 3), rather than the punctate, peripheral localization during the presence of FtsY (Supplementary Movie 2 and Figure 8A). Note that following depletion of FtsY, cells were elongated and became bent and twisted, as it has also been reported for *E. coli* (Burk et al., 2009).

We also visualized the subcellular localization of Ffh-mVenus molecules moving in confined motion, by projecting all tracks into a standardized cell of 3 × 1 μm size, roughly the average size of exponentially growing *S. putrefaciens* cells. Tracks were sorted into those that do not leave a radius of 120 nm for at least 9 steps (confined motion, red tracks), those that move freely (blue tracks), and those that show transitions between confined and free motion (green tracks). Under normal growth conditions, confined tracks were largely oriented toward the periphery of the cell, away from the cell middle, while after depletion, this preferential localization was largely lost (Figures 8C,D), in agreement with loss of interaction between SRP and the cell membrane via FtsY interaction.

### Visualization of the Preferred Subcellular Location of Distinct Mobility Fractions Shows That Putative SRP/FtsY/ribosome Transition States Occur in the Cytosol, As Well As at the Cell Membrane

**FIGURE 9:**
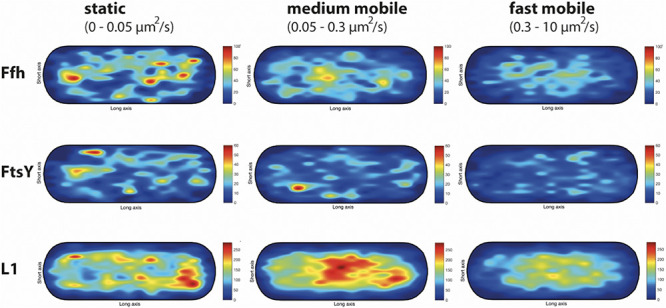
Heat maps of the preferred localization of the three distinct populations of molecules. Localization heat maps with the localization of the tracks according to their apparent diffusion D* from MSD plots, where MSD(△t) = 4⋅*D*^∗^△t. Linear fit was applied to the first 4 points of the MSD curve. A minimum of Pearson R squared of 0.8 was required. Among conditions, all heat maps have the same number of tracks (in brackets below the protein name) with approximately the same amount of detections (noted as N in the X axis label). Static fraction = D between 0 and 0.05 μm^2^/s, medium mobile = D between 0.05 and 0.3 μm^2^/s, high mobile = D 0.3–10 μm^2^/s. Colors from blue to red indicate increases in occupancy of tracks. Cells have a size of 1 × 3 μm.

We wished to analyze if the three proposed diffusive populations occupy different subcellular locations, and importantly, if the intermediate population has a preferential positioning at the cell membrane, as would be expected for the slow mobile/actively translating fraction, or if a putative SRP/FtsY/ribosome NC complex can form within the cytosol. We generated speed maps of Ffh, FtsY, and L1, considering the apparent diffusion D^∗^ (Rocha et al., 2018; Banaz et al., 2019) obtained from the linear fit of MSD curves on the first 4 time points (t = 1…,4∙Δt). Only high-quality linear fits (R^2^ ≥ 0.8) were considered for the analysis. From apparent diffusion analyses and the diffusion coefficients obtained in the GMM analyses, we found that the slow mobile/static fraction occupies a range of 0–0.05 μm^2^/s, the medium mobility fraction a range of 0.05–0.3 μm^2^/s, and the high mobility population a range of 0.3–10 μm^2^/s. According to this classification, we constructed maps of the probability of the three fractions of tracks to occur within a standardized cell of 1 × 3 μm size. Figure 9 shows that slow mobile fractions of all three proteins clustered near the cell membrane, most pronounced for ribosomes. High mobility populations (lower panels) were distributed throughout the cells, while the medium mobile molecules (average 0.13 μm^2^/s) showed an intermediate pattern (Figure 9). For freely diffusing molecules, no preference for any subcellular site would be expected, as is the case, except that the putative free 50S subunits showed a preference of diffusion toward the cell center; this can be explained by the fact that the cell is a tube. Medium mobile ribosomes could be found close to the cell membrane as well as in the cytosol, which is also true, albeit less apparent, for Ffh and FtsY.

These experiments show that SRP, FtsY, and ribosomes diffuse with a distinct, medium mobility throughout the cell, possibly forming a complex (with SRP meeting ribosomes before FtsY joins in), and then diffuse to the cell membrane for their interaction with the translocon. Medium-mobile populations are also found close to or at the cell membrane, and thus putative transition complexes might also form directly at the membrane.

## Discussion

It is textbook knowledge that SRP interacts with ribosomes and the hydrophobic nascent chains (NCs) at the peptide exit channel, and guides the ribosome/NC complex to the membrane, where it meets its receptor FtsY that in turns hands over the complex to the Sec translocon. Several recent reports have suggested that mRNA, ribosomes, and SRP might meet at the membrane, and search for FtsY by 2D diffusion, rather than by 3D diffusion from the cytosol (Prilusky and Bibi, 2009; Nevo-Dinur et al., 2011; Benhalevy et al., 2017). In order to address this important question by a cell biological approach, we sought to study SRP dynamics in live cells using super resolution fluorescence microscopy and single molecule tracking. Using the Gram negative bacterium *S. putrefaciens* as a model organism, we generated functional fluorescent protein fusions of the SRP protein component Ffh, of SRP receptor FtsY, and of L1 protein from the large ribosomal subunit. We succeeded in obtaining such fusions expressed from the respective original gene locus under native expression conditions, and were thus able to visualize dynamics of SRP, which we have not been able to find in the present literature. Using SIM, which can capture slow mobile or static molecules, we found overlapping but distinguishable localization patterns for Ffh and FtsY compared to that of ribosomes. The latter showed a pronounced accumulation in DNA-free spaces at the cell poles, while the predominantly membrane-localized Ffh and FtsY did not show polar accumulation but were randomly distributed along the cell membrane, in agreement with the idea that only a subset of ribosomes are engaged in translation of membrane proteins (Randall and Hardy, 1977; Herskovits and Bibi, 2000). Interestingly, single molecule tracking strongly suggested the existence of three distinguishable mobility fractions for Ffh/SRP and also for its receptor and for ribosomes. A slow mobile (almost static) fraction likely corresponds to actively translating polysomes, and of ribosomes in complex with SRP and FtsY at the cell membrane. Of note, we cannot distinguish between these two fractions, of which ribosome NC/SRP/FtsY complexes are a minority. The determined diffusion constants agree with data obtained from the Elf group (Sanamrad et al., 2014). The existence of high mobility fractions for all three proteins/complexes, each with distinct diffusion constants, agrees with the idea of free 50S subunits, and free SRP complexes and FtsY molecules, diffusing through the entire cell. Indeed, speed maps we devised for this study showed that the proteins fusions with the highest mobility diffuse through the entire cells with no spatial preference. Interestingly, we found a third fraction of molecules having an intermediate mobility, with very similar diffusion constants for all three proteins. In the absence of further data, the nature of these fractions remains obscure. In the meantime, we would like to propose that the medium-mobility fraction seen for L1 comprises transitory forms of the large subunit, for example 50S ribosome subunits on their transition to/and 70S initiation complexes engaged with the Shine-Dalgarno (SD) sequence at the 5′ end of mRNA, and that the transition to the slow mobile state is the subsequent binding of additional ribosomes to the mRNA, which then slows down/immobilizes mRNA at the cell poles. For a 70S ribosome engaged in translation of an mRNA for a membrane protein, binding of SRP would slow down/arrest translation until the translocon has been found. With regard to SRP, a medium-mobility population can be explained by the formation of a mobile ribosome NC/SRP/FtsY complex, for which the mobility almost entirely depends on that of the ribosome and bound mRNA due to their large size. Thus, the mobility of this complex could be very similar to that of 70S ribosome/mRNA complexes (the binding of SRP and FtsY to 70S/mRNA complexes will not lead to a considerable increase in the stokes radius of the particles). We cannot distinguish between mobile mRNA-bound ribosomes (if they exist) and mobile ribosome NC/SRP/FtsY complexes, but our findings are in agreement with many SRP as well as FtsY molecules spending a large time in a diffusive state together with ribosome NCs.

An important aspect of this study was to investigate the three-dimensional path of formation of a ribosome NC complex bound to SRP and to FtsY, which will finally be handed over to the Sec translocon. By localizing the three distinct mobility fractions, we found that as expected, the slow-mobile fraction of translating ribosomes (as well as many SRP and FtsY molecules) clustered at the cell membrane, while the fast-mobile fraction likely comprising the freely diffusing subunits were distributed throughout the cytosol (i.e., the highest concentration was in the cell middle/center). For the ribosome, this is in agreement with data showing that 50 and 30S subunits of ribosomes can diffuse through the nucleoids, while 70S subunits are largely excluded and cluster at the cell poles (Sanamrad et al., 2014). Interestingly, the medium-mobility fraction of all three proteins showed intermediate localization patterns, with more proteins being close to the cell membrane compared to the freely diffusive population, but a large fraction being cytosolic. If a part of this population is comprised of ribosomes engaged in SRP/FtsY binding, our findings would suggest that SRP binds to ribosome/NC complexes within the cytosol (Figure 1), and also at the cell membrane, after which FtsY also binds. If this was true, ribosome NCs/SRP and FtsY would diffuse to the cell membrane in 3D motion, or along the cell membrane, where they likely switch into a 2D search mode for translocons to deliver the ribosome/NCs.

Ffh orthologs from *S. putrefaciens* and the model organism *E. coli* share the same length in their primary sequences with 77% identity and 87% conservation at the amino acid level. FtsY from *S. putrefaciens* and *E. coli* share 57 and 70% identity and conservation at the amino acid level, respectively. All functional relevant motifs and elements, which have been identified for Ffh and FtsY in the well-studied model bacterium *E. coli*, are conserved in *S. putrefaciens* used in this study. Thus, domain structures and sequences are conserved between the proteins from the two species; however, differences in the SRP cycle may exist nevertheless.

Our findings suggest that subpopulations of ribosomes, of SRP and its receptor FtsY, preferentially localize to different parts of bacterial cells. Ribosomes of low mobility, which have earlier been observed by epifluorescence microscopy (Lewis et al., 2000; Mascarenhas et al., 2001), occupy spaces outside nucleoids, i.e., DNA free spaces at the cell poles, while a diffusive population earlier found by SMT studies (Sanamrad et al., 2014) moves throughout the cells. A large population with an intermediate diffusion constant, never observed before, can be found within the cytosol as well as at the cell membrane. Likewise, SRP and FtsY show membrane-proximal low mobility fractions, and medium-mobility fractions that cluster in the cytosol and at the cell membrane. While we cannot yet clearly assign functions to the SRP and FtsY fractions, it is intriguing to see that the bacterial machinery for insertion of membrane proteins into the cell membrane apparently operates in a much more intricate manner than appreciated before.

## Materials and Methods

### Bacterial Strains, Growth Conditions, and Media

*Escherichia coli* and *Shewanella putrefaciens* strains used in this study are listed in Table 3. *Shewanella* strains were grown in LB medium at room temperature or 30°C, *Escherichia coli* in LB medium at 37°C. For the 2,6-diaminopimelic acid (DAP)-auxotroph *E. coli* WM 3064 media were supplemented with DAP at a final concentration of 300 μM. Selective media were supplemented with 50 μg/ml kanamycin or 10% (w/v) sucrose, as appropriate. To solidify media 1.5% (w/v) LB agar was added. To prepare agarose pads for fluorescence microscopy, LM100 (10 mM HEPES, pH 7.3; 100 mM NaCl; 100 mM KCl; 0.02% yeast extract; 0.01% peptone; 15 mM lactate) or LB medium was solidified by adding 1% (w/v) agarose. To inhibit transcription in the mid-exponential phase (OD_600_ 0.5) the cells were treated with 25 μg/ml rifampicin. For translation inhibition the cells in mid-exponential phase were treated with 50 μg/ml chloramphenicol or 200 μg/ml puromycin.

**TABLE 3:** Bacterial strains.

**Organism**	**Relevant genotype or description**	**References**
***Escherichia coli***		
DH5αλpir	Φ80d*lacZ* ΔM15 Δ(*lacZYA-argF*)U169 *recA1 hsdR17 deoR thi-l supE44 gyrA96 relA1*/λpir	Miller and Mekalanos, 1988
WM3064	*thrB1004 pro thi rpsL hsdS lacZ*ΔM15 RP4−1360 Δ(*araBAD*) 567Δ*dapA* 1341::[*erm pir*(wt)]	*
***Shewanella putrefaciens***		
S271	CN-32, wild-type	Fredrickson et al., 1998
S4000	Ara sfGFP Δ*araDA*; ΔSputcn32_2066-2067; markerless insertion of *sfgfp* downstream of *araB* in the *ΔaraDA* deletion mutant using the remaining *ΔaraD* ATG as start codon and markerless deletion of *araDA*	This study
S4002	Ara LuxCDABE Δ*araDA*; ΔSputcn32_2066-2067; markerless insertion of *luxCDABE* of *Photorhabdus luminescens* downstream of *araB* in the *ΔaraDA* deletion mutant using the remaining *ΔaraD* ATG as start codon and markerless deletion of *araDA*	This study
S5801	Ffh-mVenus; Sputcn32_1167-3x-Gly-Ser-mVenus; markless insertion of *ffh* with a C-terminal mVenus-tag	This study
S5798	FtsY-mVenus; Sputcn32_0289-Gly-Ser-mVenus; markless insertion of *ftsY* with a C-terminal mVenus-tag	This study
S5797	RplA-mVenus; Sputcn32_3769-3xGly-Ser-sfGFP; markless insertion of *rplA* with a C-terminal mVenus-tag	This study
S5977	Ffh-sfGFP; Sputcn32_1167-3x-Gly-Ser-sfGFP; markless insertion of *ffh* with a C-terminal sfGFP-tag	This study
S6559	FtsY-sfGFP; Sputcn32_0289-Gly-Ser-sfGFP; markless insertion of *ftsY* with a C-terminal sfGFP-tag	This study
S6728	RplA-sfGFP; Sputcn32_3769-3xGly-Ser-sfGFP; markless insertion of *rplA* with a C-terminal sfGFP-tag	This study
S6482	Ara ind. FtsY-FLAG Δ*araDA* Δ*ftsY* in strain S5801 (Ffh-mVenus); Sputcn32_0289 ΔSputcn32_2066-2067; markerless insertion of *ftsY* with a C-terminal FLAG-tag downstream of *araB* in the *ΔaraDA* deletion mutant using the remaining *ΔaraD* ATG as start codon and markerless deletion of *ftsY*	This study
S5486	FtsY-FLAG; Sputcn32_0289-FLAG; markless insertion of *ftsY* with a C-terminal FLAG-tag	This study

*W. Metcalf, University of Illinois, Urbana−Champaign.

### Plasmid and Strain Constructions

Generally, genetic manipulations were carried out within the genome of *S. putrefaciens* at the corresponding native gene loci. In-frame deletions or chromosomal integrations of genes encoding fluorescently or FLAG-tagged proteins were introduced by sequential homologous recombination. For this purpose, the suicide vector pNPTS-138-RKT was used as previously described (Lassak et al., 2010). The plasmids were conjugated into *Shewanella* via *E. coli* WM3064 as a donor. All plasmids and oligonucleotides used in this study are listed in Supplementary Table 3. Construction of the plasmids was carried out using Gibson assembly (Gibson et al., 2009). All enzymes (Fermentas) and kits for purification of nucleic acids (VWR International GmbH) were used according to manufacturer protocols. To generate markerless in-frame deletions, 500–600 bp fragments of the up- and downstream region of the target gene were combined to create a deletion leaving only six codons of the 5′- and 3′-termini of the corresponding gene. The monomeric fluorescent proteins mVenus and sfGFP were fused C-terminally to the appropriate gene products with a small flexible linker.

### Employing the L-Arabinose Utilization Locus as Inducible Gene Expression System in *S. putrefaciens*

*S. putrefaciens* possesses two alternative routes of L-arabinose utilization (Rodionov et al., 2010). The major arabinose degradation pathway depends on the L-arabinose isomerase AraA (Sputcn32_2066), the L-ribulokinase AraB (Sputcn32_2068), and the L-ribulose-phosphate epimerase AraD (Sputcn32_2067). The corresponding genes are organized in an *araBDAX* operon, which is activated by AraR in the presence of arabinose (Watanabe et al., 2006). Deletion of *araD* and *araA* (Δ*araDA*) resulted in a mutant strain unable to grow when L-arabinose was the sole source of carbon (data not shown). To determine AraR-mediated control of the operon in the presence or absence of L-arabinose, the *luxCDABE* from *Photorhabdus luminescens* (Bubendorfer et al., 2012) was integrated into the chromosome directly downstream of *araB* in the Δ*araDA* deletion strain to avoid degradation of the inducer. This was carried out in a way so that the remaining *araD* ATG start codon constituted the start codon for *luxC*. We then determined L-arabinose-dependent *luxCDABE* expression by measuring light emission relative to the OD_600_ of the cell culture in complex LB medium using a Tecan Infinite M200 plate reader (Tecan). For the measurement, 100 μl cell culture in technical triplicates was transferred into a well of a white 96-well polypropylene Microtitrer plate (Greiner). In the absence of L-arabinose, no light emission occurred and the measured relative light units (RLU) equaled that of cells not harboring *luxCDABE* (Supplementary Figure 2). In contrast, upon addition of L-arabinose light was emitted in a manner that was directly depending on the concentration of L-arabinose in the medium. To further determine if AraR-controlled expression occurs in a homo- or heterogeneous fashion, the gene encoding the fluorophore sfGFP was placed into the chromosome downstream of *araB* in the Δ*araDA* mutant strain in the same fashion as described above for *luxCDABE*. sfGFP production in the presence and absence of L-arabinose was then determined by fluorescence microscopy, revealing highly homologous fluorescence in all cells in the presence of L-arabinose and no fluorescence in its absence (Supplementary Figure 2). Thus, the arabinose system provides an excellent gene expression system for *S. putrefaciens*, which is very tightly controlled and allows moderate gene induction evenly distributed among the population. Genes of interest can be integrated into the chromosome directly downstream of *araB* in the Δ*araDA* deletion mutant using the remaining *araD* ATG as start codon. Thus, this system also allowed for the first time efficient depletion experiments of (essential) proteins in *S. putrefaciens*, which we accordingly used in this study.

To this end, *ftsY* with an addition sequence adding a C-terminal FLAG-tag to the protein was integrated downstream of *araB* accordingly. Addition of 0.1% (w/v) L-arabinose in the medium yielded FtsY production levels highly similar to that of the wild type as shown by comparison with a strain bearing a FLAG-tagged version of FtsY. Then, the native *ftsY* gene (Sputcn32_0289) was deleted from the chromosome in the presence of 0.1% L-arabinose. To perform depletion experiments, the appropriated strain was inoculated from an overnight culture containing 0.1% (w/v) L-arabinose into LB-media without L-arabinose and analyzed in the midexponential growth phase at an OD_600_ of 0.5.

### Immunoblot Analysis

Production and stability of the fusions were determined by Western blot analyses. Protein lysates were prepared from exponentially growing cultures. Collection of protein samples, protein separation, and immunoblot detection were essentially carried out as described earlier (Bubendorfer et al., 2012; Schuhmacher et al., 2015). To detect the proteins monoclonal, horseradish peroxidase-conjugated antibody raised against the FLAG-tag (Sigma Aldrich) in the dilution of 1:1,000 and polyclonal antibody raised against GFP or mVenus (Roche) in the dilution of 1:5,000 were used. Secondary anti-mouse IgG-alkaline phosphatase antibody was used at a dilution of 1:5,000 to detect GFP antibody. Signals were detected with SuperSignal^TM^ West Pico PLUS Chemiluminescent Substrate (Thermo Scientific) or CDP-Star chemiluminescent substrate (Roche Diagnostics) and were documented using a Fusion-SL chemiluminescent imager (Peqlab).

### Epifluorescence Microscopy

*Shewanella* strains were cultured to mid-exponential phase before imaging. A total of 2.5 μl of the culture was spotted on an LB medium-agarose pad. Fluorescence images to analyze the homogeneous expression of sfGFP of the L-arabinose inducible system were recorded with a DMI6000B inverse microscope (Leica) equipped with a pco.edge sCMOS camera (PCO) using the VisiView software (Visitron Systems GmbH). Images were further processed using ImageJ 1.52v software (National Institutes of Health) and Affinity Designer 1.7v (Serif).

### Structured Illumination Microscopy

Samples at mid-exponential phase were mounted on ultrapure-agarose slides dissolved in LB (1%) for immobilization of cells prior to image acquisition. For localization experiments, image Z-stacks (∼100 nm steps) were acquired using brightfield (BF) image acquisition (transmitted light) or structured illumination microscopy (SIM) with a ZEISS ELYRA PS.1 setup (Andor EMCCD camera, 160 nm pixel size; 3× rotations and 5× phases per z-slice; grating period: 42 μm; 200 mW laser line (between 80 and 200 W/cm^2^) at excitation laser wavelength 488 nm; ZEISS alpha Plan-Apochromat 100x/NA 1.46 Oil DIC M27 objective). SIM reconstructions were processed using ZEN-Black software by ZEISS. ImageJ2/FIJI version 1.52p was used for visualization and image processing (Linkert et al., 2010; Schindelin et al., 2012; Schneider et al., 2012; Rueden et al., 2017). Region(s) of interest (ROI) were defined by cell borders using the brush-selection tool to maintain good contrast levels of cellular areas. SIM reconstructions were manually cropped in axial and lateral dimensions, depending on plausibility of cellular positions, using “Duplicate”-function. Signal located outside cell borders was considered to be background and was therefore eliminated. Resulting image z-stacks were projected using FIJI implemented “Z-project”-function (e.g., “Average Intensity”), false-colored and color-balance adjusted to generate tomographic representations. ROI of fluorescence micrographs were limited by cell borders. Artifacts outside ROI (e.g., dust particles on camera) were manually corrected. 3D SIM image z-stacks movies were visualized using FIJI implemented 3D-Project function (with interpolation) for 360° visualization and z-stacks for a tomographic walk-through. Resulting 3D-visualizations were generated with merged channels, processed and transformed as.avi movies, and finally combined in a sequential manner using FIJI.

### Single Particle Tracking

Slimfield microscopy underfills the back aperture of the objective by reducing the width of the laser beam, generating an area of about 10 μm diameter containing high light intensity allowing visualization of single fluorescent proteins at very high acquisition rates. Single molecule level is obtained by bleaching of most molecules in the cell (between 100 and 1,000 frames), followed by tracking of remaining and newly synthesized molecules for about 3,000 frames. A 16 ms stream acquisition was used on Olympus IX-71 or Nikon Ti eclipse microscopes (Objective 100x, NA 1.49), frames were captured by an Andor iXON Ultra 987 EMCCD camera using Andor Solis program, or using a Hamamatsu ImageEM X2 camera and Nikon NIS software. Freely diffusive proteins such as GFP or monomeric fluorescent proteins can only be accurately followed by very fast acquisition rates in the low milliseconds range, as rapid movement during longer acquisition times leads to blurring of the signal. Because we wanted to analyze movement of larger proteins (FtsY) and of protein/RNA complexes, we used an acquisition rate of 16 ms, which is able to capture movement of freely diffusive large proteins (Sanamrad et al., 2014). We used YFP-bleaching SPT, which avoids blue light toxicity (El Najjar et al., 2020), and employed laser power densities between 150 and 200 W cm^–1^ that allow for continued growth of cells after acquisition (cells were also mounted on LB agarose pads). Cell borders were defined using program Oufti (Paintdakhi et al., 2016), resulting tracking data were generated using utrack (Jaqaman et al., 2008) (the centroid position of each identified molecule is statistically fitted providing localization precision of less than 50 nm) and analyzed using SMTracker (Rosch et al., 2018), which involves several statistical tests (see Table 1) to determine if acquired distributions of molecule movements can be explained by single or multiple fractions. SMTracker uses a Bayesian Information Criterion-based algorithm to avoid over-fitting of data.

## Data Availability Statement

The original contributions presented in the study are included in the article/Supplementary Material, further inquiries can be directed to the corresponding author/s.

## Author Contributions

BM performed all experiments and analyzed data shown in Figures 1–9 and Supplementary Figure 2, and helped in writing of the manuscript. MS constructed the Shewanella depletion system and performed experiments shown in Supplementary Figures 1, 3, 4. LO-B devised the tool for visualization of different diffusive populations shown in Figure 9 and helped analyze data. GB helped design the study and in writing of the manuscript. KT devised the Shewanella depletion strain and supervised corresponding experiments, devised the study, and helped writing the manuscript. PG devised of the study and supervised all Bacillus experiments, helped analyze the data, and wrote the manuscript. All authors contributed to the article and approved the submitted version.

## Conflict of Interest

The authors declare that the research was conducted in the absence of any commercial or financial relationships that could be construed as a potential conflict of interest.

## Supplementary Material

Supplementary Figure 1 Stable fluorescent fusion proteins. **(A)** Western blot depicting stable fluorescent fusion of mVenus and sfGFP with FtsY, SRP, and ribosomes (L1). Coomassie gel act as loading control. **(B)** Western blot depicting stable fluorescent fusions proteins after different time points of chloramphenicol (Cm) treatment.

Supplementary Figure 2 GMM analyses of FlrA during exponential growth (steady state) and 60 min after addition of puromycin (stress). Right panels shows diffusion constants and fraction sizes with error bars as indicated in the legend.

Supplementary Figure 3 L-arabinose inducible gene expression system in *S. putrefaciens*. **(A)** Luciferase assay depicting well-inducible *luxCDABE* expression by integration into the L-arabinose utilization gene locus downstream of *araB* using the *araD* ATG as start codon. Relative light units (RLU) are given as mean value ± standard deviation of three biological replicates and three technical replicates. **(B)** Homogeneous expression of sfGFP in the L-arabinose utilization gene locus by addition of 0.2% L-arabinose. Scale bar 5 μm.

Supplementary Figure 4 Depletion of FtsY. **(A)** Western blot depicting the depletion of FtsY-FLAG to less than 10% of wild type (wt) level in absence of L-arabinose. Coomassie gel act as loading control. **(B)** Western blot depicting stable Ffh-mVenus fusion by depleted FtsY. **(C)** Growth curve of *S. putrefaciens* FtsY depletion strain without and in the presence of 0.1% L-arabinose in LB medium. OD_600_ is given as mean value ± standard deviation of two independent measurements.

Supplementary Table 1 GMM analyses of drug treatments.

Supplementary Table 2 GMM analyses of FtsY depletion.

Supplementary Table 3 Plasmids and oligonucleotides.

Supplementary Movie 1 Stream acquisition (16 ms) of *Shewanella putrefaciens* cells expressing Ffh-mVenus during exponential growth. First frame shows bright field acquisition, following processed frames YFP fluorescence. Movie speed 50 fps (almost real time speed).

Supplementary Movie 2 3D SIM reconstruction for Ffh-sfGFP p*ara*-FtsY in *S. putrefaciens*. Cells were grown in the presence of arabinose, such that FtsY is expressed at physiological levels. Z-stacks resulting from bright field acquisition and sfGFP channels are merged and projected into tomographic representations. Fluorescent emissions are false-colored in “thal” showing particle densities from low (blue) to high (red). Movie Speed 12 fps.

Supplementary Movie 3 3D SIM reconstruction for Ffh-sfGFP p*ara*-FtsY in *S. putrefaciens*. Cells were grown in the absence of arabinose, such that FtsY is expressed at 10% of its physiological levels. Z-stacks resulting from bright field acquisition and sfGFP channels are merged and projected into tomographic representations. Fluorescent emissions are false-colored in “thal” showing particle densities from low (blue) to high (red). Movie Speed 12 fps.
