# Supplementary figures and images for: Dynamics of Bacterial Signal Recognition Particle at a Single Molecule Level
Source: Front Microbiol. 2021 Apr 30;12:663747. doi: 10.3389/fmicb.2021.663747 (PMC8120034; doi:10.3389/fmicb.2021.663747)

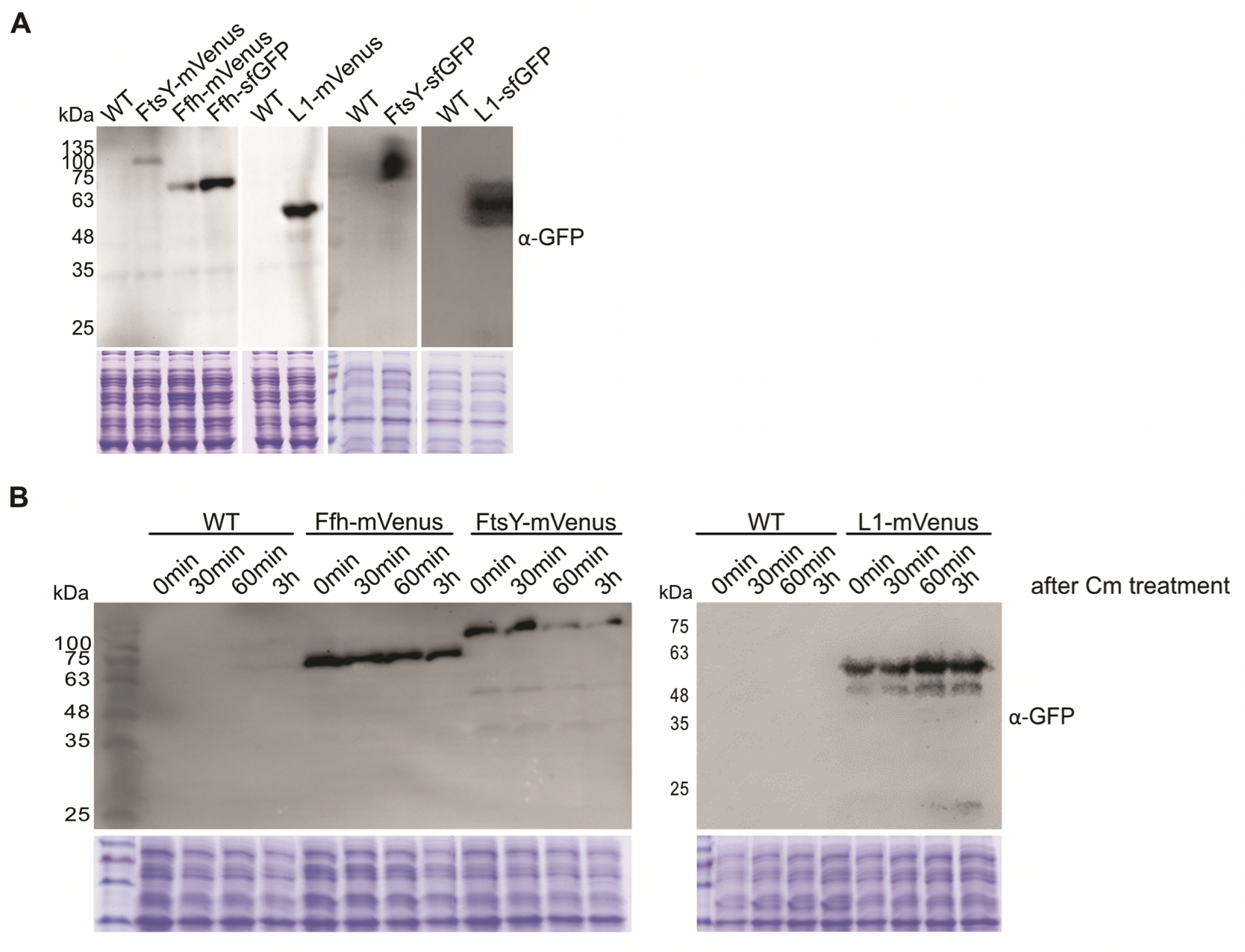

Supplement: Supplementary Figure 1 — Stable fluorescent fusion proteins. (A) Western blot depicting stable fluorescent fusion of mVenus and sfGFP with FtsY, SRP, and ribosomes (L1). Coomassie gel act as loading control. (B) Western blot depicting stable fluorescent fusions proteins after different time points of chloramphenicol (Cm) treatment. [file Image_1.TIFF]

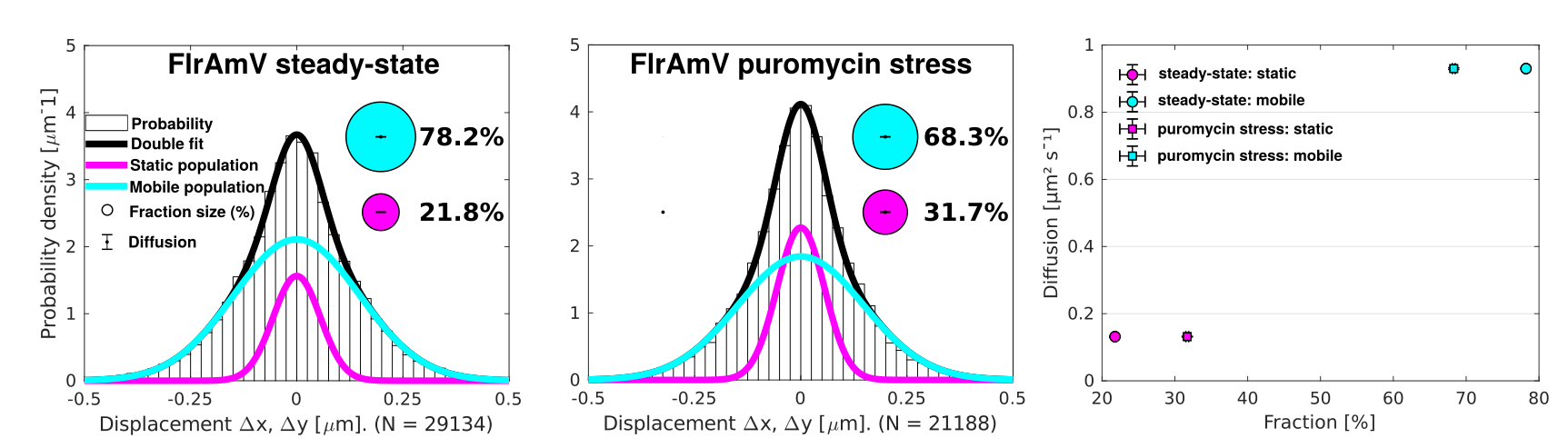

Supplement: Supplementary Figure 2 — GMM analyses of FlrA during exponential growth (steady state) and 60 min after addition of puromycin (stress). Right panels shows diffusion constants and fraction sizes with error bars as indicated in the legend. [file Image_2.TIFF]

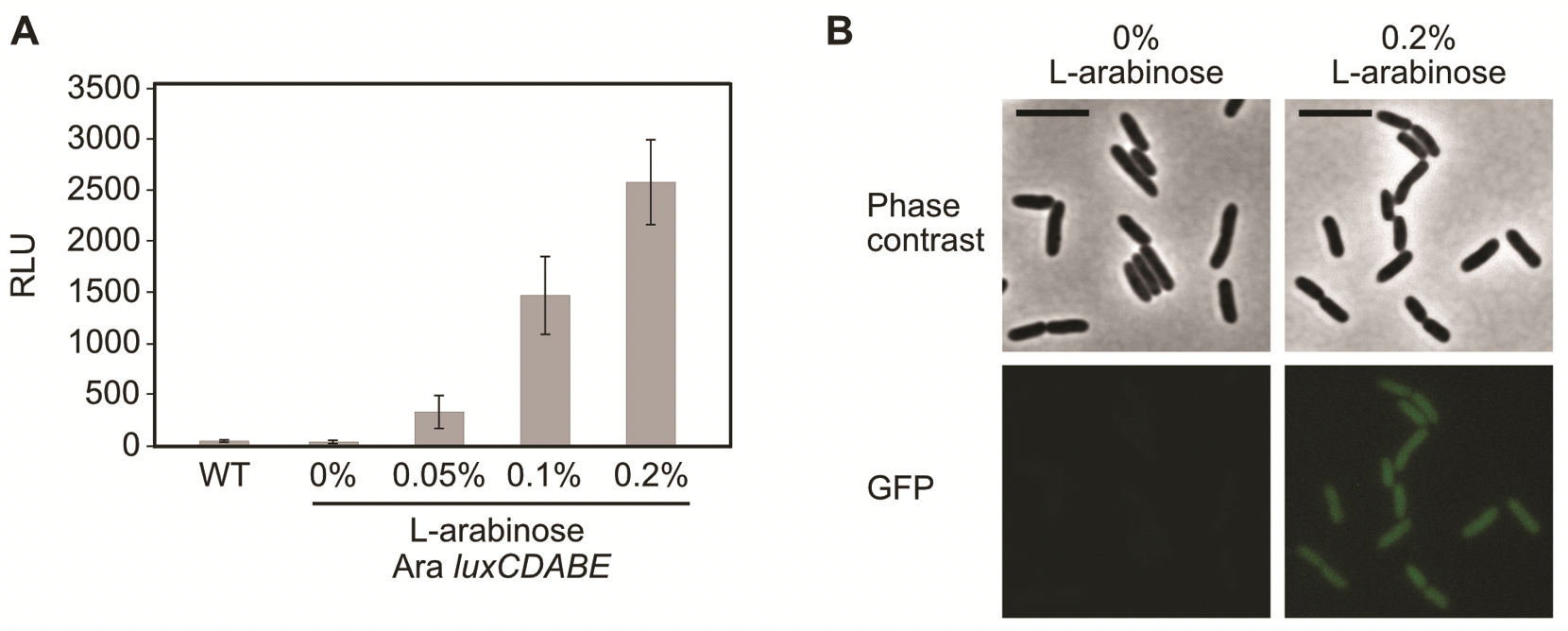

Supplement: Supplementary Figure 3 — L-arabinose inducible gene expression system in S. putrefaciens. (A) Luciferase assay depicting well-inducible luxCDABE expression by integration into the L-arabinose utilization gene locus downstream of araB using the araD ATG as start codon. Relative light units (RLU) are given as mean value ± standard deviation of three biological replicates and three technical replicates. (B) Homogeneous expression of sfGFP in the L-arabinose utilization gene locus by addition of 0.2% L-arabinose. Scale bar 5 μm. [file Image_3.TIFF]

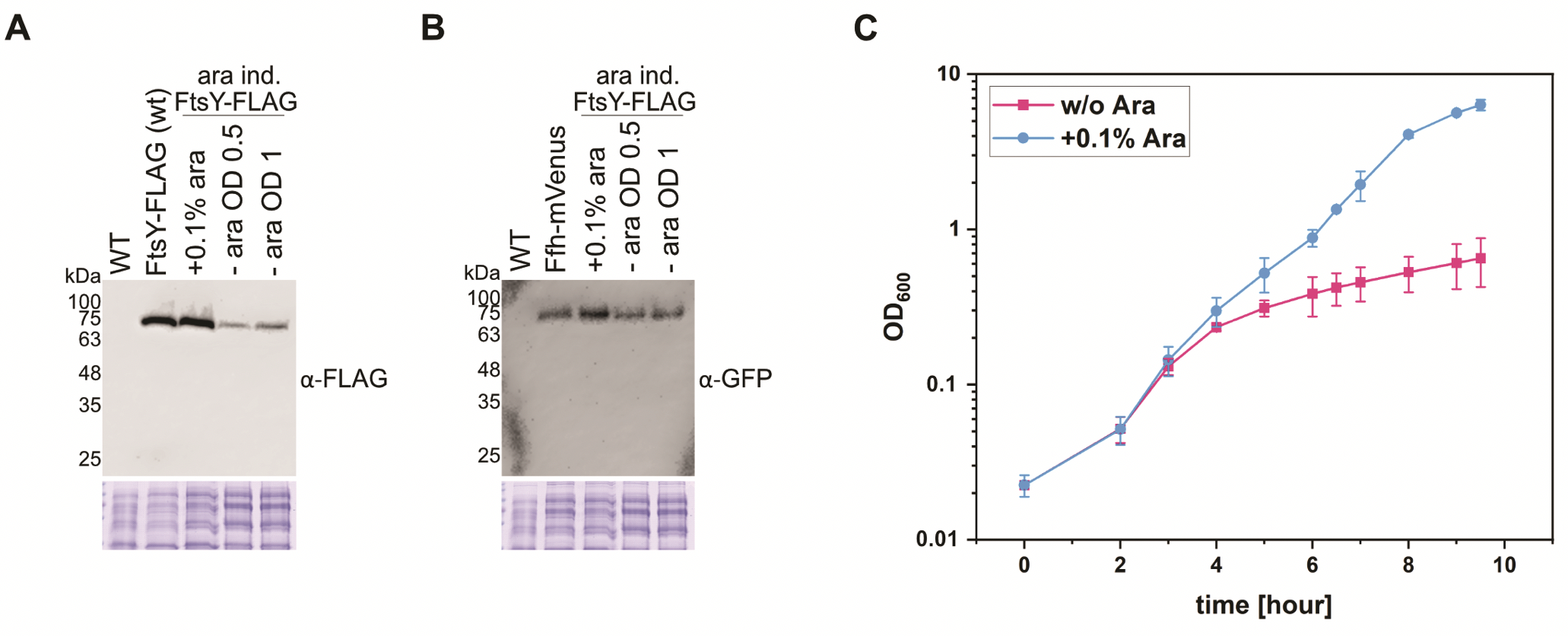

Supplement: Supplementary Figure 4 — Depletion of FtsY. (A) Western blot depicting the depletion of FtsY-FLAG to less than 10% of wild type (wt) level in absence of L-arabinose. Coomassie gel act as loading control. (B) Western blot depicting stable Ffh-mVenus fusion by depleted FtsY. (C) Growth curve of S. putrefaciens FtsY depletion strain without and in the presence of 0.1% L-arabinose in LB medium. OD600 is given as mean value ± standard deviation of two independent measurements. [file Image_4.TIFF]
