# Supplementary material for: Dynamics of Bacterial Signal Recognition Particle at a Single Molecule Level
Source: Front Microbiol. 2021 Apr 30;12:663747. doi: 10.3389/fmicb.2021.663747 (PMC8120034; doi:10.3389/fmicb.2021.663747)
Supplement: Supplementary Table 1 — GMM analyses of drug treatments. [file Table_1.DOCX]

**Table S1 GMM analyses of drug treatments**

**Rifampicin treatment**

**Ffh steady state after 30 min after 60 min**

| # movies | 14 | 15 | 14 |  |
| --- | --- | --- | --- | --- |
| # cells | 63 | 46 | 61 |  |
| av. cell length [µm] | 2.0500 | 2.7700 | 2.2000 |  |
| # tracks | 768 | 519 | 541 |  |
| av. lifetime [frames / s] | 5.6 / 0.099 | 5.7 / 0.1 | 5.5 / 0.098 |  |
| Static D ± SD [µm² sˉ¹] | 0.025 ± 0.0021 | 0.025 ± 0.0021 | 0.025 ± 0.0021 | |
| Slow-mobile D ± SD [µm² sˉ¹] | 0.14 ± 0.007 | 0.14 ± 0.007 | 0.14 ± 0.007 | |
| Mobile D ± SD [µm² sˉ¹] | 0.92 ± 0.023 | 0.92 ± 0.023 | 0.92 ± 0.023 | |
| Static Fraction ± SD [%] | 16 ± 1.5 | 5.2 ± 0.89 | 4 ± 0.76 | |
| Slow-mobile Fraction ± SD [%] | 47 ± 1.2 | 47 ± 0.96 | 43 ± 1.1 | |
| Mobile Fraction ± SD [%] | 37 ± 1.3 | 48 ± 0.93 | 53 ± 0.92 | |
| K-S GoF* test | Accepted | Accepted | Accepted | |
| P-Value | 0.99 | 0.6 | 0.78 | |
| R-Squared | 1 | 1 | 1 | |
| Best Model | Triple Fit | Triple Fit (*) | Triple Fit (*) | |

**FtsY steady state after 30 min after 60 min**

| # movies | 12 | 10 | 10 |  |
| --- | --- | --- | --- | --- |
| # cells | 33 | 68 | 48 |  |
| av. cell length [µm] | 2.4800 | 2.1500 | 2.6000 |  |
| # tracks | 125 | 306 | 243 |  |
| av. lifetime [frames / s] | 5.7 / 0.1 | 5.4 / 0.097 | 5 / 0.089 |  |
| Static D ± SD [µm² sˉ¹] | 0.027 ± 0.008 | 0.027 ± 0.008 | 0.027 ± 0.008 | |
| Slow-mobile D ± SD [µm² sˉ¹] | 0.14 ± 0.025 | 0.14 ± 0.025 | 0.14 ± 0.025 | |
| Mobile D ± SD [µm² sˉ¹] | 0.72 ± 0.034 | 0.72 ± 0.034 | 0.72 ± 0.034 | |
| Static fraction ± SD [%] | 23 ± 8.2 | 5.4 ± 3.2 | 3.9 ± 1.8 | |
| Slow-mobile fraction ± SD [%] | 58 ± 6.9 | 37 ± 2.7 | 18 ± 3.6 | |
| Mobile fraction ± SD [%] | 19 ± 7.6 | 57 ± 2.9 | 78 ± 2.7 | |
| K-S GoF test | Accepted | Accepted | Accepted | |
| P-Value | 0.89 | 0.59 | 0.9 | |
| R-Squared | 0.999 | 0.999 | 1 | |
| Best Model | Triple Fit (*) | Triple Fit (*) | Triple Fit (*) | |

**L1 steady state after 30 min after 60 min**

| # movies | 5 | | 5 | 1 |  |
| --- | --- | --- | --- | --- | --- |
| # cells | 14 | | 23 | 2 |  |
| av. cell length [µm] | 2.2200 | | 2.5200 | 4.6500 |  |
| # tracks | 2353 | | 3161 | 251 |  |
| av. lifetime [frames / s] | 6.5 / 0.1 | | 6.3 / 0.1 | 5.5 / 0.088 |  |
| Static D ± SD [µm² sˉ¹] | 0.056 ± 0.0005 | | 0.056 ± 0.0005 | 0.056 ± 0.0005 | |
| Slow-mobile D ± SD [µm² sˉ¹] | 0.32 ± 0.006 | | 0.32 ± 0.006 | 0.32 ± 0.006 | |
| Mobile D ± SD [µm² sˉ¹] | 0.96 ± 0.014 | | 0.96 ± 0.014 | 0.96 ± 0.014 | |
| Static fraction ± SD [%] | 34 ± 0.31 | | 3.5 ± 0.16 | 0.1 ± 0.27 | |
| Slow-mobile fraction ± SD [%] | 40 ± 0.69 | 51 ± 1.1 | | 21 ± 1.7 | |
| Mobile fraction ± SD [%] | 26 ± 0.5 | 46 ± 0.64 | | 79 ± 0.97 | |
| K-S GoF test | Accepted | Accepted | | Accepted | |
| P-Value | 0.76 | 0.87 | | 0.64 | |
| R-Squared | 1 | 1 | | 1 | |
| Best Model | Triple Fit | Triple Fit | | Triple Fit | |

**Chloramphenicol**

**Ffh steady state after 30 min after 60 min**

| # movies | | 8 | 9 | | | 9 | |
| --- | --- | --- | --- | --- | --- | --- | --- |
| # cells | | 91 | 59 | | | 47 | |
| av. cell length [µm] | | 2.3100 | 2.7900 | | | 3.2900 | |
| # tracks | | 1857 | 1064 | | | 2199 | |
| av. lifetime [frames / s] | | 6.3 / 0.11 | 5.7 / 0.1 | | | 6 / 0.11 | |
| Static D ± SD [µm² sˉ¹] | 0.047 ± 0.0007 | | | 0.047 ± 0.0007 | 0.047 ± 0.0007 | |  |
| Slow-mobile D ± SD [µm² sˉ¹] | 0.18 ± 0.01 | | | 0.18 ± 0.01 | 0.18 ± 0.01 | |  |
| Mobile D ± SD [µm² sˉ¹] | 0.82 ± 0.019 | | | 0.82 ± 0.019 | 0.82 ± 0.019 | |  |
| Static Fraction ± SD [%] | 59 ± 1.2 | | | 19 ± 0.9 | 13 ± 0.64 | |  |
| Slow-mobile Fraction ± SD [%] | 25 ± 1.2 | | | 40 ± 0.96 | 34 ± 1.2 | |  |
| Mobile Fraction ± SD [%] | 17 ± 1.2 | | | 40 ± 0.93 | 53 ± 0.93 | |  |
| K-S GoF test | Accepted | | | Accepted | Accepted | |  |
| P-Value | 0.28 | | | 0.27 | 0.68 | |  |
| R-Squared | 1 | | | 1 | 1 | |  |
| Best Model | Triple Fit | | | Triple Fit | Triple Fit | |  |

**FtsY steady state after 30 min after 60 min**

| # movies | 13 | | 13 | 13 |  |
| --- | --- | --- | --- | --- | --- |
| # cells | 169 | | 137 | 123 |  |
| av. cell length [µm] | 2.6800 | | 3.1400 | 3.4800 |  |
| # tracks | 2427 | | 3221 | 3168 |  |
| av. lifetime [frames / s] | 5.7 / 0.1 | | 6 / 0.11 | 5.9 / 0.1 |  |
| Static D ± SD [µm² sˉ¹] | 0.027 ± 0.00028 | 0.027 ± 0.00028 | | 0.027 ± 0.00028 | |
| Slow-mobile D ± SD [µm² sˉ¹] | 0.14 ± 0.0024 | 0.14 ± 0.0024 | | 0.14 ± 0.0024 | |
| Mobile D ± SD [µm² sˉ¹] | 0.67 ± 0.0058 | 0.67 ± 0.0058 | | 0.67 ± 0.0058 | |
| Static Fraction ± SD [%] | 33 ± 0.4 | 18 ± 0.31 | | 13 ± 0.24 | |
| Slow-mobile Fraction ± SD [%] | 35 ± 0.35 | 43 ± 0.33 | | 38 ± 0.4 | |
| Mobile Fraction ± SD [%] | 31 ± 0.37 | 39 ± 0.32 | | 48 ± 0.32 | |
| K-S GoF test | Accepted | Accepted | | Accepted | |
| P-Value | 0.65 | 0.82 | | 0.94 | |
| R-Squared | 1 | 1 | | 1 | |
| Best Model | Triple Fit | Triple Fit | | Triple Fit | |

**L1 steady state after 30 min after 60 min**

| # movies | 5 | 5 | 5 | |  |
| --- | --- | --- | --- | --- | --- |
| # cells | 46 | 65 | 63 | |  |
| av. cell length [µm] | 2.4300 | 2.6300 | 3.2000 | |  |
| # tracks | 9724 | 17911 | 26561 | |  |
| av. lifetime [frames / s] | 6.8 / 0.12 | 6.7 / 0.12 | 7.2 / 0.13 | |  |
| Static D ± SD [µm² sˉ¹] | 0.032 ± 5.3e-05 | 0.032 ± 5.3e-05 | | 0.032 ± 5.3e-05 | |
| Slow-mobile D ± SD [µm² sˉ¹] | 0.13 ± 0.00038 | 0.13 ± 0.00038 | | 0.13 ± 0.00038 | |
| Mobile D ± SD [µm² sˉ¹] | 0.83 ± 0.0017 | 0.83 ± 0.0017 | | 0.83 ± 0.0017 | |
| Static fraction ± SD [%] | 21 ± 0.073 | 31 ± 0.084 | | 32 ± 0.088 | |
| Slow-mobile fraction ± SD [%] | 47 ± 0.05 | 44 ± 0.056 | | 45 ± 0.057 | |
| Mobile fraction ± SD [%] | 31 ± 0.062 | 25 ± 0.07 | | 23 ± 0.073 | |
| K-S GoF test | Accepted | Accepted | | Accepted | |
| P-Value | 0.54 | 0.99 | | 0.95 | |
| R-Squared | 1 | 1 | | 1 | |
| Best Model | Triple Fit | Triple Fit | | Triple Fit | |

**Puromycin**

**Ffh steady stat after 30 min after 60 min**

| # movies | 29 | 31 | | 43 | | |
| --- | --- | --- | --- | --- | --- | --- |
| # cells | 125 | 170 | | 134 | | |
| av. cell length [µm] | 2.6900 | 3.3400 | | 3.8300 | | |
| # tracks | 6305 | 7299 | | 7633 | | |
| av. lifetime [frames / s] | 6.6 / 0.12 | 6.2 / 0.11 | | 6.6 / 0.12 | | |
| Static D ± SD [µm² sˉ¹] | 0.046 ± 0.0003 | | 0.046 ± 0.0003 | | 0.046 ± 0.0003 |  |
| Slow-mobile D ± SD [µm² sˉ¹] | 0.19 ± 0.002 | | 0.19 ± 0.002 | | 0.19 ± 0.002 |  |
| Mobile D ± SD [µm² sˉ¹] | 0.86 ± 0.006 | | 0.86 ± 0.006 | | 0.86 ± 0.006 |  |
| Static Fraction ± SD [%] | 35 ± 0.26 | | 19 ± 0.19 | | 8.1 ± 0.16 |  |
| Slow-mobile Fraction ± SD [%] | 36 ± 0.28 | | 44 ± 0.29 | | 50 ± 0.31 |  |
| Mobile Fraction ± SD [%] | 29 ± 0.27 | | 38 ± 0.24 | | 42 ± 0.24 |  |
| K-S GoF test | Accepted | | Accepted | | Accepted |  |
| P-Value | 0.71 | | 0.093 | | 0.72 |  |
| R-Squared | 1 | | 1 | | 1 |  |
| Best Model | Triple Fit | | Triple Fit | | Triple Fit |  |

**FtsY steady state after 30 min after 60 min**

| # movies | 33 | 37 | | 35 |  |
| --- | --- | --- | --- | --- | --- |
| # cells | 138 | 157 | | 95 |  |
| av. cell length [µm] | 2.8100 | 3.5500 | | 4.0300 |  |
| # tracks | 2995 | 3965 | | 3033 |  |
| av. lifetime [frames / s] | 6.3 / 0.11 | 5.9 / 0.11 | | 6.1 / 0.11 |  |
| Static D ± SD [µm² sˉ¹] | 0.031 ± 0.0003 | | 0.031 ± 0.0003 | 0.031 ± 0.0003 | |
| Slow-mobile D ± SD [µm² sˉ¹] | 0.15 ± 0.0021 | | 0.15 ± 0.0021 | 0.15 ± 0.0021 | |
| Mobile D ± SD [µm² sˉ¹] | 0.75 ± 0.0076 | | 0.75 ± 0.0076 | 0.75 ± 0.0076 | |
| Static fraction ± SD [%] | 27 ± 0.36 | | 11 ± 0.24 | 14 ± 0.27 | |
| Slow-mobile fraction ± SD [%] | 43 ± 0.34 | | 50 ± 0.38 | 49 ± 0.35 | |
| Mobile fraction ± SD [%] | 30 ± 0.35 | | 39 ± 0.31 | 37 ± 0.31 | |
| K-S GoF test | Accepted | | Accepted | Accepted | |
| P-Value | 0.98 | | 0.66 | 0.27 | |
| R-Squared | 1 | | 1 | 1 | |
| Best Model | Triple Fit | | Triple Fit | Triple Fit | |

**L1 steady after 30 min after 60 min**

| # movies | 29 | 22 | 26 |  |
| --- | --- | --- | --- | --- |
| # cells | 161 | 106 | 94 |  |
| av. cell length [µm] | 2.8300 | 3.4800 | 3.7500 |  |
| # tracks | 32131 | 25407 | 24283 |  |
| av. lifetime [frames / s] | 9.4 / 0.17 | 9.2 / 0.16 | 9 / 0.16 |  |
| Static D ± SD [µm² sˉ¹] | 0.044 ± 5.4e-05 | 0.044 ± 5.4e-05 | 0.044 ± 5.4e-05 | |
| Slow-mobile D ± SD [µm² sˉ¹] | 0.17 ± 0.0003 | 0.17 ± 0.0003 | 0.17 ± 0.0003 | |
| Mobile D ± SD [µm² sˉ¹] | 0.66 ± 0.0008 | 0.66 ± 0.0008 | 0.66 ± 0.0008 | |
| Static fraction ± SD [%] | 27 ± 0.056 | 19 ± 0.05 | 15 ± 0.041 | |
| Slow-mobile fraction ± SD [%] | 41 ± 0.041 | 46 ± 0.041 | 44 ± 0.049 | |
| Mobile fraction ± SD [%] | 32 ± 0.048 | 35 ± 0.045 | 41 ± 0.045 | |
| K-S GoF test | Accepted | Accepted | Accepted | |
| P-Value | 0.49 | 0.98 | 0.52 | |
| R-Squared | 1 | 1 | 1 | |
| Best Model | Triple Fit | Triple Fit | Triple Fit | |

**FlrA steady state after 60 min**

| # movies | 20 | 17 |
| --- | --- | --- |
| # cells | 130 | 89 |
| av. cell length [µm] | 2.8100 | 3.6300 |
| # tracks | 2783 | 2080 |
| av. lifetime [frames / s] | 6.2 / 0.073 | 6.1 / 0.072 |
| *Double fit* |  |  |
| Static D ± SD [µm² sˉ¹] | 0.13 ± 0.001 | 0.13 ± 0.001 |
| Mobile D ± SD [µm² sˉ¹] | 0.93 ± 0.0026 | 0.93 ± 0.0026 |
| Static fraction ± SD [%] | 22 ± 0.18 | 32 ± 0.2 |
| Mobile fraction ± SD [%] | 78 ± 0.18 | 68 ± 0.2 |
| K-S GoF test | Accepted | Accepted |
| P-Value | 0.51 | 0.58 |
| R-Squared | 1 | 1 |

*Kolmogorov-Smirnov Goodness-of-Fit Test
