# Supplementary material for: Dynamics of Bacterial Signal Recognition Particle at a Single Molecule Level
Source: Front Microbiol. 2021 Apr 30;12:663747. doi: 10.3389/fmicb.2021.663747 (PMC8120034; doi:10.3389/fmicb.2021.663747)
Supplement: Supplementary Table 2 — GMM analyses of FtsY depletion. [file Table_2.DOCX]

**Table S2 GMM analyses of FtsY depletion**

|  | Plus arabinose | Minus arabinose |
| --- | --- | --- |
| Static D ± SD [µm² sˉ¹] | 0.029 ± 6.3e-05 | 0.029 ± 6.3e-05 |
| Slow-mobile D ± SD [µm² sˉ¹] | 0.16 ± 0.00036 | 0.16 ± 0.00036 |
| Mobile D ± SD [µm² sˉ¹] | 1.1 ± 0.0008 | 1.1 ± 0.0008 |
| Static fraction ± SD [%] | 17 ± 0.043 | 13 ± 0.04 |
| Slow-mobile fraction ± sd [%] | 36 ± 0.032 | 38 ± 0.03 |
| Mobile fraction ± sd [%] | 47 ± 0.038 | 49 ± 0.035 |
| K-S GoF test | Accepted | Accepted |
| P-Value | 0.47 | 0.096 |
| R-Squared | 1 | 1 |
| Best Model | Triple Fit | Triple Fit |

*Kolmogorov-Smirnov Goodness-of-Fit Test
