# Supplementary material for: Dynamics of Bacterial Signal Recognition Particle at a Single Molecule Level
Source: Front Microbiol. 2021 Apr 30;12:663747. doi: 10.3389/fmicb.2021.663747 (PMC8120034; doi:10.3389/fmicb.2021.663747)
Supplement: Supplementary Table 3 — Plasmids and oligonucleotides. [file Table_3.DOCX]

| **Table S3: Plasmids and oligonucleotides** | | | |  | |  |  |
| --- | --- | --- | --- | --- | --- | --- | --- |
|  | | |  |  | |  |  |
| **Name** | | | **Insert** | **Purpose** | | **Reference** |  |
|  | | |  |  | |  |  |
| pNPTS138-R6KT | | | *mob*RP4+, *ori*-R6K, *sacB,* beta-galactosidase fragment alpha, Km^r^ | suicide plasmid for in-frame deletions or integrations | | 1 |  |
| pNPTS138-R6KT-Ara ind. *luxCDABE* | | | *luxCDABE* | in-frame insertion fragment | | This study |  |
| pNPTS138-R6KT-Ara ind. *sfgfp* | | | *sfgfp* | in-frame insertion fragment | | This study |  |
| pNPTS138-R6KT-Ara ind. *araAD* KO | | | Δ*araAD* (Sputcn32_2066, Sputcn32_2067) | in-frame deletion fragment | | This study |  |
| pNPTS138-R6KT *ffh*-3xGS-mVenus | | | *ffh*-3xGS-mVenus (Sputcn32_1167) | in-frame insertion fragment, C-terminal mVenus-tagged version of *ffh* | | This study |  |
| pNPTS138-R6KT *ftsY*-GS-mVenus | | | *ftsY*-GS-mVenus (Sputcn32_0289) | in-frame insertion fragment, C-terminal mVenus-tagged version of *ftsY* | | This study |  |
| pNPTS138-R6KT *rplA*-3xGS-mVenus | | | *rplA*-3xGS-mVenus (Sputcn32_3769) | in-frame insertion fragment, C-terminal mVenus-tagged version of *rplA* | | This study |  |
| pNPTS138-R6KT *ffh*-3xGS-sfGFP | | | *ffh*-3xGS-sfGFP (Sputcn32_1167) | in-frame insertion fragment, C-terminal sfGFP-tagged version of *ffh* | | This study |  |
| pNPTS138-R6KT *ftsY*-GS-sfGFP | | | *ftsY*-GS-sfGFP (Sputcn32_0289) | in-frame insertion fragment, C-terminal sfGFP-tagged version of *ftsY* | | This study |  |
| pNPTS138-R6KT *rplA*-3xGS-sfGFP | | | *rplA*-3xGS-sfGFP (Sputcn32_3769) | in-frame insertion fragment, C-terminal sfGFP-tagged version of *rplA* | | This study |  |
| pNPTS138-R6KT Ara ind. *ftsY*-FLAG_AraAD KO | | | *ftsY*-FLAG (Sputcn32_0289); Δ*araAD* (Sputcn32_2066-2067) | in-frame insertion and deletion fragment, insertion of C-terminal FLAG-tagged version of *ftsY* and deletion of *araAD* | | This study |  |
| pNPTS138-R6KT *ftsY* KO | | | Δ*ftsY* (Sputcn32_0289) | in-frame deletion fragment | | This study |  |
| pNPTS138-R6KT *ftsY*-FLAG | | | *ftsY*-FLAG (Sputcn32_0289) | in-frame insertion fragment, C-terminal FLAG-tagged version of *ftsY* | | This study |  |
|  |  |  | | |  | | |
| **Number** | **Name** | **Sequence 5'-3'** | | | **Purpose** | | |
|  |  |  | | |  | | |
| MS91 | EcoRV-ffh venus-fwd | GCG AAT TCG TGG ATC CAG ATC CAC ATT ACG GGC AAG CCT ATT | | | construction of in-frame insertion vector pNPTS138-R6KT-*ffh-*3xGS*-*mVenus | | |
| MS92 | OL-Ffh venus-rev | AGC TCC TCG CCC TTG CTC ACG CTG CCG CTG CCG CTG CCA CGT CCT GGA AAT TTC ATA CCG | | |  |  |  |
| MS79 | OL_venus fwd | GTG AGC AAG GGC GAG GAG CT | | |  |  |  |
| MS80 | OL_venus rev | TTA CTT GTA CAG CTC GTC CAT GCC | | |  |  |  |
| MS93 | OL-Ffh-venus-fwd | GGC ATG GAC GAG CTG TAC AAG TAA TCC TAC CTA TTT TTA CTA AAG | | |  |  |  |
| MS94 | EcoRV-Ffh venus-rev | GCC AAG CTT CTC TGC AGG ATG CAG TTG CTT TAC GAG CGT CTT | | |  |  |  |
| MS95 | Check-Ffh venus-fwd | CGA TGA CTT CCA AGG AGC GTA A | | | check primer for *ffh* | | |
| MS96 | Check-Ffh venus-rev | CTG GCG ATT ATT AAA CCT GCC C | | |  |  |  |
| MS103 | EcoRV-ftsY venus-fwd | GCG AAT TCG TGG ATC CAG ATG GTA AGT CTG TGA TGT TAG CCG | | | construction of in-frame insertion vector pNPTS138-R6KT-*ftsY-*GS*-*mVenus | | |
| MS104 | OL-ftsY venus-rev | AGC TCC TCG CCC TTG CTC ACG CTG CCG TTA TCC GCT TTT TCT TGA GTG | | |  |  |  |
| MS79 | OL_venus fwd | GTG AGC AAG GGC GAG GAG CT | | |  |  |  |
| MS80 | OL_venus rev | TTA CTT GTA CAG CTC GTC CAT GCC | | |  |  |  |
| MS105 | OL-ftsY-venus-fwd | GGC ATG GAC GAG CTG TAC AAG TAA TTT ATG ATT GAT TTT CAG CAG G | | |  |  |  |
| MS106 | EcoRV-ftsY-venus-rev | GCC AAG CTT CTC TGC AGG ATC CCG CGT CAT TGA AGG TTT CAA | | |  |  |  |
| MS107 | Check-ftsY-venus-fwd | GGG TGG TGT GGT ATT TGC GAT T | | | check primer for *ftsY* | | |
| MS108 | Check-ftsY-venus-rev | CGA ACA CAC TAC GGT CCA TCA A | | |  |  |  |
| MS77 | EcoRV-rplA-venus-fwd | GCG AAT TCG TGG ATC CAG ATT GGT GCA ACT GTG TTA CCA CAC | | | construction of in-frame insertion vector pNPTS138-R6KT-*rplA-3x*GS*-*mVenus | | |
| MS78 | OL-rplA-venus-rev | AGC TCC TCG CCC TTG CTC ACG CTG CCG CTG CCG CTG CCG TTA GCT GTA TCG AGA GTC GCT | | |  |  |  |
| MS79 | OL_venus fwd | GTG AGC AAG GGC GAG GAG CT | | |  |  |  |
| MS80 | OL_venus rev | TTA CTT GTA CAG CTC GTC CAT GCC | | |  |  |  |
| MS81 | OL-rplA-venus-fwd | GGC ATG GAC GAG CTG TAC AAG TAA TTT TAC AAG GTG AGC GCA TTA G | | |  |  |  |
| MS82 | EcoRV-rplA-venus-rev | GCC AAG CTT CTC TGC AGG ATG TGA ACG TTT CTG CTA GGC ACT | | |  |  |  |
| MS83 | Check-rplA venus-fwd | GGC TTT ACT CCT GGA TTC TAC C | | | check primer for *rplA* | | |
| MS84 | Check-rplA venus-rev | TAA GCA TCT CCA CCA CTA TGG G | | |  |  |  |
| MS91 | EcoRV-ffh venus-fwd | GCG AAT TCG TGG ATC CAG ATC CAC ATT ACG GGC AAG CCT ATT | | | construction of in-frame insertion vector pNPTS138-R6KT-*ffh-*3xGS*-*sfGFP | | |
| MS240 | OL-Ffh sfGFP-rev | GAA AAG TTC TTC TCC TTT GCT GCT GCC GCT GCC GCT GCC ACG TCC TGG AAA TTT CAT ACC G | | |  |  |  |
| FR393 | OL_-m_gfp_fw | AGC AAA GGA GAA GAA CTT TTC | | |  |  |  |
| FR412 | OL_gfp_rv | GGA TCC TTT GTA GAG CTC ATC C | | |  |  |  |
| MS241 | OL-Ffh-sfGFP-fwd | GGA TGA GCT CTA CAA AGG ATC CTA ATC CTA CCT ATT TTT ACT AAA G | | |  |  |  |
| MS94 | EcoRV-Ffh-venus-rev | GCC AAG CTT CTC TGC AGG ATG CAG TTG CTT TAC GAG CGT CTT | | |  |  |  |
| MS103 | EcoRV-ftsY venus-fwd | GCG AAT TCG TGG ATC CAG ATG GTA AGT CTG TGA TGT TAG CCG | | | construction of in-frame insertion vector pNPTS138-R6KT-*ftsY-*GS*-*sfGFP | | |
| MS465 | OL-ftsY sfGFP-rev | GAA AAG TTC TTC TCC TTT GCT GCT GCC GTT ATC CGC TTT TTC TTG AGT G | | |  |  |  |
| FR393 | OL_-m_gfp_fw | AGC AAA GGA GAA GAA CTT TTC | | |  |  |  |
| FR412 | OL_gfp_rv | GGA TCC TTT GTA GAG CTC ATC C | | |  |  |  |
| MS466 | OL-ftsY-GFP-fwd | GGA TGA GCT CTA CAA AGG ATC CTA ATT TAT GAT TGA TTT TCA GCA GG | | |  |  |  |
| MS106 | EcoRV-ftsY-venus-rev | GCC AAG CTT CTC TGC AGG ATC CCG CGT CAT TGA AGG TTT CAA | | |  |  |  |
| MS77 | EcoRV-rplA-venus-fwd | GCG AAT TCG TGG ATC CAG ATT GGT GCA ACT GTG TTA CCA CAC | | | construction of in-frame insertion vector pNPTS138-R6KT-*rplA*-3xGS-sfGFP | | |
| MS463 | OL-rplA-GFP-rev | GAA AAG TTC TTC TCC TTT GCT GCT GCC GCT GCC GCT GCC GTT AGC TGT ATC GAG AGT CGC T | | |  |  |  |
| FR393 | OL_-m_gfp_fw | AGC AAA GGA GAA GAA CTT TTC | | |  |  |  |
| FR412 | OL_gfp_rv | GGA TCC TTT GTA GAG CTC ATC C | | |  |  |  |
| MS464 | OL-rplA-GFP-fwd | GGA TGA GCT CTA CAA AGG ATC CTA ATT TTA CAA GGT GAG CGC ATT AG | | |  |  |  |
| MS82 | EcoRV-rplA-venus-rev | GCC AAG CTT CTC TGC AGG ATG TGA ACG TTT CTG CTA GGC ACT | | |  |  |  |
| JH402 | EcoRV-2068-fwd | GCG AAT TCG TGG ATC CAG ATT TGA AGA TGA AAC CTT AGC AAA ACT CG | | | construction of in-frame insertion vector pNPTS138-R6KT-Ara ind. *ftsY*-FLAG Δ*araAD* | | |
| MS363 | OL_2068_FtsY-rev | CTT TGC CAT ATT ATT TCG CTC CAT CAA CAT AAT GGC | | |  |  |  |
| MS364 | OL_2068_FtsY-fwd | CGA AAT AAT ATG GCA AAG AAA GGT TTT TTC TCT TGG | | |  |  |  |
| MS365 | OL_FtsY-Flag-rev | AAT ATC ATG ATC TTT ATA ATC GCC ATC ATG ATC TTT ATA ATC GTT ATC CGC TTT TTC TTG AGT G | | |  |  |  |
| MS361 | OL_Flag-AraAD-KO-fwd | ATT ATA AAG ATC ATG ATA TTG ATT ATA AAG ATG ATG ATG ATA AAT TAA CCT TCT TAA GTT AAG AGC CGC C | | |  |  |  |
| MS362 | OL_AraAD-KO-rev-EcoRV-rev | GCC AAG CTT CTC TGC AGG ATG TTT GGG TTC ACT GAC CTG ATC | | |  |  |  |
| JH408 | Check-Ara indu.2068-fwd | GGT TGG TGC CAA TGT TAA AGC G | | | check primer for Ara ind. *ftsY*-FLAG Δ*araAD* | | |
| JH409 | Check-Ara indu.2068-rev | AAC GTA ACA GGT AAT TTT CAT AAA GCC | | |  |  |  |
| MS370 | EcoRV-FtsY KO-fwd | GCG AAT TCG TGG ATC CAG ATC CAT CAT TGA GCC AAC CTT GAG | | | construction of in-frame deletion vector pNPTS138-R6KT-Δ*ftsY* | | |
| MS371 | OL FtsY KO-rev | GTT ATC CGC CTT TGC CAT GTG TGA TTC CAA CGC | | |  |  |  |
| MS372 | OL FtsY KO-fwd | ATG GCA AAG GCG GAT AAC TAA TTT ATG ATT GAT T | | |  |  |  |
| MS106 | EcoRV-ftsY-venus-rev | GCC AAG CTT CTC TGC AGG ATC CCG CGT CAT TGA AGG TTT CAA | | |  |  |  |
| MS373 | Check-ftsY KO fwd | GGA TGG CGA GTT GCG TTA ATT C | | | check primer for Δ*ftsY* | | |
| MS108 | Check-ftsY-venus-rev | CGA ACA CAC TAC GGT CCA TCA A | | |  |  |  |
| DD1 | EcoRV-FtsY-rev | GCG AAT TCG TGG ATC CAG ATG CCA AGG TAA GTC TGT GAT GTT | | | construction of in-frame deletion vector pNPTS138-R6KT-*ftsY*-FLAG | | |
| DD2 | OL-2069_FtsY-fwd | AAT ATC ATG ATC TTT ATA ATC GCC ATC ATG ATC TTT ATA ATC GTT ATC CGC TTT TTC TTG AGT G | | |  |  |  |
| DD3 | OL-2068-FtsY-FLAG-rev | ATT ATA AAG ATC ATG ATA TTG ATT ATA AAG ATG ATG ATG ATA AAT AAT TTA TGA TTG ATT TTC AGC AGG TC | | |  |  |  |
| DD4 | EcoRV-FtsY-fwd | GCC AAG CTT CTC TGC AGG ATG CAA GTA GCA ATG GTG GCT TGT | | |  |  |  |
| DD5 | Check-FtsY-FLAG rev | CGA GTA CAG GCC AAA ATG CGA T | | | check primer for *ftsY*-FLAG | | |
| JH142 | Check-FlgK1-FLAG-fwd | GAT CTT TAT AAT CGC CAT CAT GAT C | | |  |  |  |
| SH663 | CN32 Ara Lux up fw | GCG AAT TCG TGG ATC CAG AT CCG TTT AGC CGT AGA ACA TGA TTC | | | construction of in-frame insertion vector pNPTS138-R6KT-Ara ind. *luxCDABE* | | |
| SH664 | CN32 Ara Lux up rev | CAT TAG CAT CCC CGT ACA TTG AAC | | |  |  |  |
| SH665 | CN32 Ara Lux ins fw | AAT GTA CGG GGA TGC TAA TGG GAG ACC GCG GTC CCG AAT T | | |  |  |  |
| SH667 | CN32 Ara Lux ins rev | CGT AAG ACC CTA AAA CAT TAG CTC AAC TAT CAA ACG CTT CGG TTA AG | | |  |  |  |
| SH668 | CN32 Ara Lux dwn fw | GCT AAT GTT TTA GGG TCT TAC GCG | | |  |  |  |
| SH669 | CN32 Ara Lux dwn rev | GCC AAG CTT CTC TGC AGG AT GAC TTT ATC TGA AGT GCA GGT TTC C | | |  |  |  |
| SH673 | CN32 Ara check fw | CGC TTT AAC ATT GGC ACC AAC C | | | check primer for Ara | | |
| SH674 | CN32 Ara check rev | CGT GAT GAC ATG CTC AAA CTG C | | |  |  |  |
| SH663 | CN32 Ara Lux up fw | GCG AAT TCG TGG ATC CAG AT CCG TTT AGC CGT AGA ACA TGA TTC | | | construction of in-frame insertion vector pNPTS138-R6KT-Ara ind. s*fgfp* | | |
| SH664 | CN32 Ara Lux up rev | CAT TAG CAT CCC CGT ACA TTG AAC | | |  |  |  |
| SH670 | CN32 Ara sfGFP ins fw | AAT GTA CGG GGA TGC TAA TGA GCA AAG GAG AAG AAC TTT TCA CTG | | |  |  |  |
| SH672 | CN32 Ara sfGFP ins rev | CGT AAG ACC CTA AAA CAT TAG CTT AGG ATC CTT TGT AGA GCT CAT CC | | |  |  |  |
| SH668 | CN32 Ara Lux dwn fw | GCT AAT GTT TTA GGG TCT TAC GCG | | |  |  |  |
| SH669 | CN32 Ara Lux dwn rev | GCC AAG CTT CTC TGC AGG AT GAC TTT ATC TGA AGT GCA GGT TTC C | | |  |  |  |
| SH675 | CN32 AraAD KO up fw | GCG AAT TCG TGG ATC CAG AT GAC CTG CGA TAT TAC CGT TGC | | | construction of in-frame insertion vector pNPTS138-R6KT-Ara ind. *araAD* KO | | |
| SH676 | CN32 AraAD KO up rev | GGT AAA TTA TAG CCC AAA TGA CAT ATT ATT TCG CTC CAT CAA C | | |  |  |  |
| SH677 | CN32 AraAD KO dwn fw | AAT ATG TCA TTT GGG CTA TAA TTT ACC TTC TTA AGT TAA G | | |  |  |  |
| SH678 | CN32 AraAD KO dwn rev | GCC AAG CTT CTC TGC AGG AT GGG ATT TCA ACC AAA GAA AAA CAT TGG | | |  |  |  |
| SH679 | CN32 AraAD check fw | GGT TGG TGC CAA TGT TAA AGC G | | | check primer for Δ*araAD* | | |
| SH680 | CN32 AraAD check rev | AAC GTA ACA GGT AAT TTT CAT AAA GCC | | |  |  |  |

References

1. Lassak, J., Henche, A. L., Binnenkade, L. & Thormann, K. M. ArcS, the cognate sensor kinase in an atypical arc system of shewanella oneidensis MR-1. Applied and Environmental Microbiology 76, 3263–3274 (2010).
